# Polyhydroxyurethane Networks from Soybean Oil and CO_2_: Thermal Behavior, Hybrid Features, and Structural Insights

**DOI:** 10.1021/acsomega.5c09377

**Published:** 2026-03-19

**Authors:** Pedro H. M. Nicácio, Ana Beatriz S. Barros, Carlos B. B. Luna, Andreas Ries, Hidetake Imasato, Edcleide M. Araújo, Ubirajara P. Rodrigues-Filho, Renate M. R. Wellen

**Affiliations:** † Department of Materials Engineering (DEMat), Federal University of Campina Grande (UFCG), Campina Grande, Paraíba 58429-900, Brazil; ‡ Hybrid Materials Chemistry Group (GQMATHI), São Carlos Institute of Chemistry (IQSC), 61755 University of São Paulo (USP), São Carlos, São Paulo 13563-120, Brazil; § Bio and Materials Group, Polytechnic School, 187173 National University of Asunción, San Lorenzo 111421, Paraguay; ∥ Department of Materials Engineering (DEMAt), Federal University of Paraíba (UFPB), João Pessoa, Paraíba 58051-900, Brazil

## Abstract

In response to increasing global demand for biobased polymers, this study presents the synthesis and comprehensive thermal characterization of nonisocyanate polyurethanes (NIPUs) derived from soybean oil, focusing on the identification of volatile compounds released during thermal analysis and proposing a degradation mechanism. The materials were obtained via epoxidation, CO_2_ cycloaddition, and aminolysis using different amines—IPDA, TRIS, and APTES—forming poly­(hydroxyurethanes) (PHUs) with variable cross-link densities and hybrid features. Structural analyses via ^1^H NMR and FTIR confirmed successful conversion at each step, with epoxy group titration indicating ≈91% conversion to cyclic carbonate and efficient catalyst removal, as confirmed by XRF (Br < 0.3% after purification). PHUs achieved high cross-linking, with gel contents up to 91.9%. The presence of APTES significantly modified network architecture, reducing gel content to 33% in some formulations but introducing inorganic siloxane domains. TGA revealed multistage degradation between 250 and 475 °C, with TRIS-based networks demonstrating enhanced thermal resistance. Deconvoluted DTG curves and TG-IR data identified primary volatiles including CO_2_, amines, ketenes, and silanols, elucidating degradation pathways involving urethane cleavage, retro-aminolysis, and glycerol decomposition. This work advances the understanding of structure–property–degradation relationships in NIPUs and highlights their viability as green engineering alternatives to conventional polyurethanes, aligning with SDGs 9, 12, and 13.

## Graphical abstract

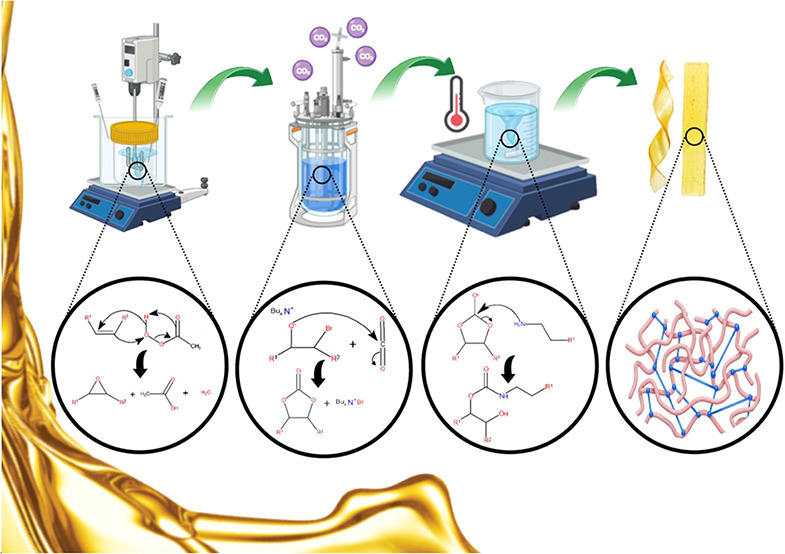

## Introduction

The growing concern regarding the environmental impacts associated with the synthetic polymers derived from fossil sources has driven the development of biobased alternatives.^1–4^ This movement is supported by international initiatives such as the United Nations Sustainable Development Goals (SDGs), particularly SDG 9 (Industry, Innovation and Infrastructure), SDG 12 (Responsible Consumption and Production), and SDG 13 (Climate Action), as well as emerging chemical technologies highlighted by IUPAC, including “reactive extrusion” and “plastics to monomers”.^5–7^

Polyurethanes (PUs), versatile materials used in foams, adhesives, coatings, and elastomers,^8,9^ represent a global market estimated at over USD 90 billion in 2024, with continued growth projections.^10–15^ However, conventional PU synthesis involves the use of diisocyanates—highly toxic petroleum-derived substances known for their adverse effects on human health, especially in occupational settings.^16,17^ In response to these concerns, the European Union implemented Regulation (EU) 2020/1149, effective since 2023, which restricts the use of diisocyanates and mandates compulsory training for exposed workers.^18^

Against this backdrop, alternative routes that eliminate the use of isocyanates are attracting increasing interest.^19,20^ Among these, nonisocyanate polyurethanes (NIPUs), synthesized via the reaction of cyclic carbonates with polyamines to form polyhydroxyurethanes (PHUs), stand out.^4,21–30^ These routes not only eliminate the toxicological risks associated with isocyanates but also provide an efficient pathway for the chemical fixation of CO_2_ into polymeric materials, promoting carbon sequestration and contributing to climate change mitigation.^31–35^

Since the 1990s, carbon dioxide (CO_2_) chemistry and its utilization as a feedstock in the synthesis of organic compounds have attracted increasing attention from the scientific community, this growing interest stems from CO_2_’s potential as an alternative carbon source—abundant, inexpensive, and nontoxic—while simultaneously offering an effective strategy for mitigating greenhouse gas emissions and, consequently, contributing to the deceleration of global warming.^33,35,36^

Previously regarded solely as a greenhouse gas associated with global warming, CO_2_ has been progressively re-envisioned as a green building block with the potential to replace fossil-based sources in material production.^34^ This approach offers a significant environmental advantage: in addition to employing CO_2_ as a feedstock, the carbon remains chemically fixed within the polymer structure. Polymers generally exhibit longer life cycles than synthetic fuels derived from CO_2_, whose emissions rapidly return to the atmosphere after combustion. Consequently, such materials act as temporary carbon reservoirs, directly contributing to climate change mitigation. Among its most promising applications is its use in the synthesis of cyclic carbonate-derived monomers, which are employed in the production of polyhydroxyurethanes (PHUs) or in the formulation of organic polycarbonates, both of which are fully aligned with the principles of green chemistry and the circular economy.^31^

The production of NIPUs from vegetable oils such as soybean oil (SO) is particularly attractive due to its abundance, low cost, high chemical functionality, and renewable nature.^4,37–39^ SO can be functionalized via epoxidation and subsequently converted into cyclic carbonates through CO_2_ cycloaddition, using catalysts such as tetrabutylammonium bromide (TBAB) under mild conditions.^40–43^ The subsequent aminolysis step with polyamines such as isophorone diamine (IPDA) or tris­(2-aminoethyl)­amine (TRIS) leads to the formation of polymeric networks with varying cross-link densities and physicochemical properties.^44–46^ Meanwhile, the addition of APTES leads to the formation of a hybrid material due to its ethoxysilane phase.^47–49^

Works have demonstrated the potential of PHUs derived from renewable sources. Poussard et al. (2016)^50^ reported thermal stability up to 350 °C for PHUs based on oligoamides. Liu et al. (2021)^51^ showed that SO-based materials retained their thermal stability after multiple reprocessing cycles. Pouladi et al. (2021)^52^ investigated linseed oil-derived PHUs and observed thermal stability up to 270 °C. Significant advances in the development of SO-based PHUs highlight their potential for high-performance applications, such as substantial improvements in mechanical properties, high degree of cross-linking, superior thermal stability, and adjustable glass transitions, which depend on the chemical structure and chain length of the amines used.^4,53–55^ Despite these advances, a comprehensive understanding of the thermal degradation mechanisms and the nature of volatile compounds evolved from these materials is still lacking, information that is essential for applications requiring high temperatures, especially under nonisothermal conditions.

Literature reports that the degradation of isocyanate-derived PUs can occur via three pathways: dissociation, transesterification, and formation of primary and secondary amines; as briefly exemplified in Figure 1.^56–59^ Moreover, studies correlating the structural characteristics of PHUs and the amines employed—such as flexibility and steric hindrance—with thermal degradation parameters and associated kinetics are scarce. Detailed understanding of these aspects is essential for optimizing material performance in industrial applications requiring thermal stability, recyclability, and aging resistance.

**Figure 1:**
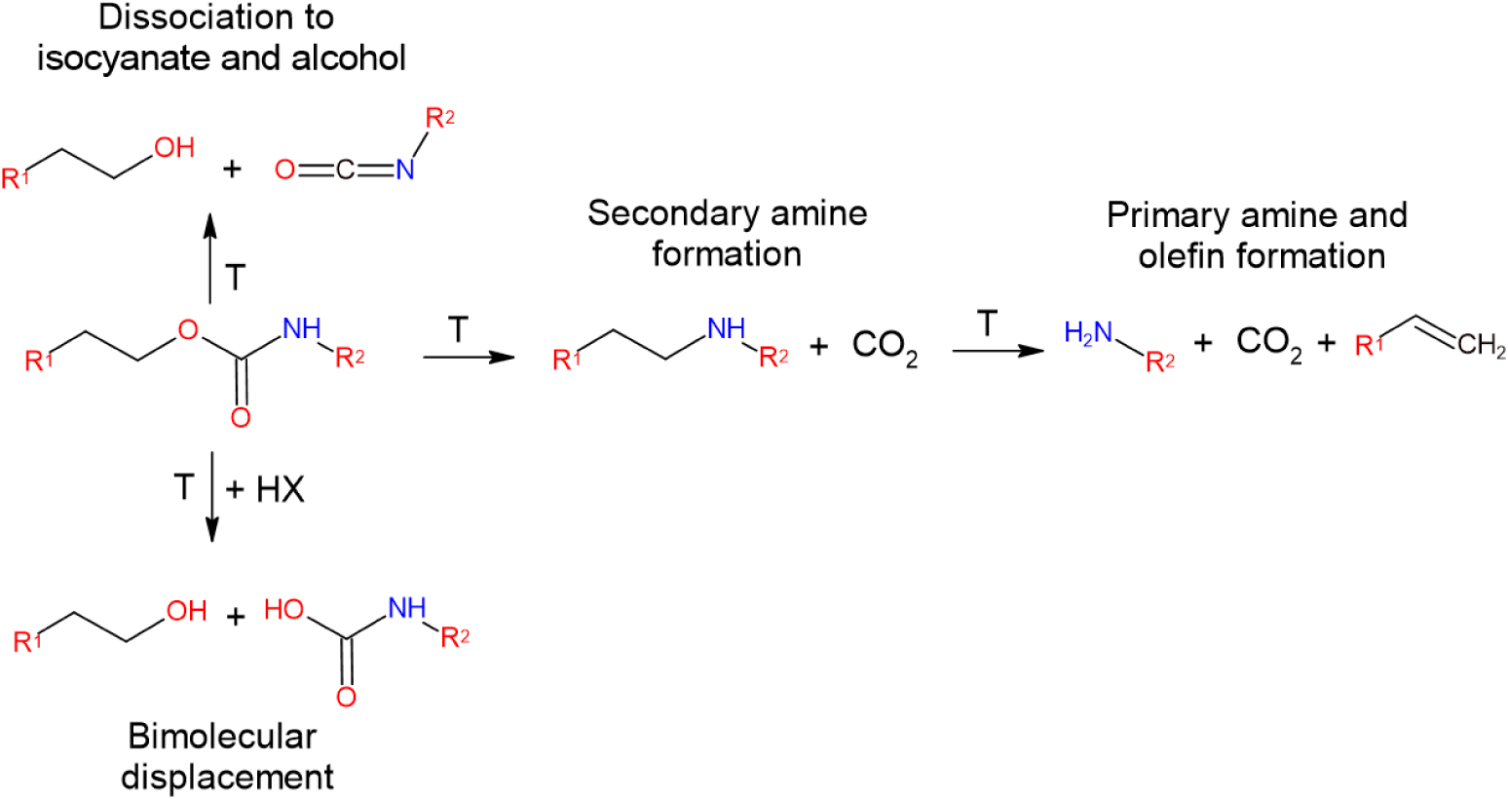
Schematic representation of the thermal degradation pathways of polyurethane, illustrating the main dissociation routes of urethane linkages under thermal activation, including bond cleavage leading to the formation of amines, alcohols, CO_2_, and olefinic fragments.

In light of this scenario, this study reports the synthesis of biobased poly­(hydroxyurethanes) (PHUs) derived from soybean oil through a sequential process comprising epoxidation, cyclocarbonation, and aminolysis, using two polyamines and an aminoalkoxysilane as reactive agents. Particular emphasis is placed on elucidating the thermal stability, degradation mechanisms, and the nature of volatile compounds generated during the thermal decomposition process, crucial factors that imply their applications under high temperatures and knowledge of compounds released during their thermal degradation. The study includes characterization techniques such as ^1^H and ^13^C NMR, XRF, FTIR, and TGA coupled with infrared spectroscopy of evolved gases (TG-IR). This combined analysis provides deeper insights into the degradation pathways and structure–property relationships, thereby contributing to the rational design of high-performance and nonisocyanate polyurethanes (NIPUs).

## Methodology

### Materials

This section describes the materials used for the synthesis of polyhydroxyurethanes (PHUs) from soybean oil, as well as the auxiliary reagents employed in the epoxidation, cyclocarbonation, and aminolysis processes.

#### Main Precursors

●Commercial soybean oil (SO), supplied by Soya (Brazil), with an average molar mass (MM) ≈ 909 g·mol^–1^ and iodine value (IV) of 129.7 g I_2_/100 g, as determined by ^1^H NMR (see Supporting Information, Table S1 and eqs 1, 2, 3 and 4).

●Epoxidised soybean oil (ESO), synthesized from SO, with MM ≈975 g·mol^–1^ and an average functionality of 4.2 epoxy groups per molecule (confirmed by ^1^H NMR and titration according to ASTM D1652–04).

●Soybean oil-based cyclic carbonate (CSBO), obtained by CO_2_ cycloaddition to ESO, with a conversion yield of ≈92%, MM ≈1151 g·mol^–1^, characterized by ^1^H NMR and epoxy titration (ASTM D1652–04).

#### Amines Used in the Aminolysis Reaction

●Isophorone diamine (IPDA), cis/trans mixture, purity ≥99%, MM = 170.3 g·mol^–1^ (Merck-Sigma-Aldrich). IPDA was selected due to its cyclic structure, which allows assessment of the impact of steric hindrance on cross-linking.

●Tris­(2-aminoethyl)­amine (TRIS), purity 96%, MM = 146.23 g·mol^–1^ (Merck-Sigma-Aldrich), chosen for its three primary amine groups and high conformational flexibility, favoring a higher cross-linking density

●(3-Aminopropyl)­triethoxysilane (APTES), purity ≥ 98%, MM = 221.37 g·mol^–1^ (Merck-Sigma-Aldrich), used as a hybrid functional agent with potential for siloxane formation via hydrolysis–condensation.

#### Auxiliary Reagents and Solvents

● Formic acid 85% (Neon, Brazil), MM = 46.03 g·mol^–1^ – precursor agent for the peracid used in the epoxidation step.

●Hydrogen peroxide 50% (Êxodo Científica, Brazil), MM = 34.01 g·mol^–1^ – oxidant for in situ peracid formation.

● Anhydrous sodium carbonate, analytical grade (Êxodo Científica), MM = 105.99 g·mol^–1^ – used for neutralization of the aqueous phase.

● Ethyl acetate 99.5% analytical grade (Neon, Brazil), MM = 88.11 g·mol^–1^ – extraction solvent.

● Tetrabutylammonium bromide (TBAB), purity ≥98%, MM = 322.4 g·mol^–1^ (Êxodo Científica) – cyclocarbonation catalyst.

● Carbon dioxide (CO_2_), purity ≥99.9%, supplied by White Martins (São Paulo, Brazil), compressed in gas cylinders – used for building block in the formation of the cyclic carbonate.

● All reagents were used as received, without further purification. Additional analyses of the fatty acid composition of soybean oil and detailed experimental data are available in the Supporting Information (Table S1 and eqs 1, 2, 3, and 4).

### Syntheses and Characterizations

#### Epoxidation of Soybean Oil (ESO)

The reaction was based on Quadros and Giudici (2016), with modifications. A total of 150 g of commercial soybean oil (SO), 16.24 g of formic acid (FA), and 102 g of hydrogen peroxide (H_2_O_2_, 50%) were used in a glass reactor with mechanical stirring (1000 rpm). The mixture was maintained at 60 ± 2 °C for 4 h. A molar ratio of 1:0.5:3 was employed, corresponding to 1 mol of soybean oil, 0.5 mol of 85% formic acid, and 3 mol of hydrogen peroxide (H_2_O_2_) per mol of soybean oil—equivalent to 6 mol of H_2_O_2_ per mol of formic acid. After the reaction, the pH was adjusted to approximately 3–4 using 0.1 N sodium carbonate. The mixture was transferred to a separatory funnel and allowed to phase-separate, thus the aqueous phase (containing polar oxidants and residual formic acid/hydrogen peroxide) was discarded. The organic phase was dissolved in ethyl acetate and the solvent was removed under reduced pressure to afford ESO. ESO was characterized by ^1^H and ^13^C NMR, FTIR, and titration (ASTM D1652-04).

#### Cyclocarbonation of ESO (CSBO)

The conversion of ESO to cyclic carbonate was performed in a 400 mL stainless steel autoclave, using 100 g (≈0.1 mol) of ESO and 8.8 g (≈0.03 mol) TBAB (catalyst). The system was purged with CO_2_, pressurized to 1.3 MPa, and maintained at 120 °C for 24 h under magnetic stirring (250 rpm). After cooling, the product was dissolved in ethyl acetate and washed six times with ultrapure water to remove the TBAB. The solvent was removed and the resulting CSBO was characterized by ^1^H and ^13^C NMR, FTIR, XRF, and epoxy titration.

#### Synthesis of PHUs

PHU formulations were prepared via stoichiometric aminolysis between amine and cyclic carbonate groups (R = 1), using different combinations of IPDA, TRIS, and APTES. CSBO was heated to 50 °C under magnetic stirring, and the amines were added according to the formulation. The samples were molded in silicone molds and cured at 80–100 °C. Conversion was monitored by FTIR (reduction of the 1796 cm^–1^ band of carbonyl (C=O) stretching from cyclic carbonate), and the absence of bubbles confirmed the absence of residual solvents. Four formulations of hydroxyurethanes derived from soybean oil cyclocarbonate were produced, as shown in Table 1.

**Table 1:** Formulations of Produced Polyhydroxyurethanes

	Materials			
PHUs	CSBO (mmol)	IPDA (mmol)	APTES (mmol)	TRIS (mmol)
**CSBO/IPDA**	2.8	5.76	-	-
**CSBO/IPDA/APTES**	2.8	5.59	0.35	-
**CSBO/TRIS**	2.8	-	-	3.84
**CSBO/TRIS/APTES**	2.8	-	0.35	3.72

#### Nuclear Magnetic Resonance Spectroscopy (NMR)

The ^1^H and ^13^C NMR spectra of SO, ESO, and CSBO were acquired on an Agilent 400 MHz, 400/54 Premium Shielded system, using 15 mg of sample in 0.6 mL of CDCl_3_, 99.8% D atom, and referenced at 7.26 ppm.

#### Epoxy Group Titration

Quantification of epoxy groups followed ASTM D1652-04,^60^ using 0.4 g of sample dissolved in dichloromethane and titrated with 0.12 N perchloric acid in the presence of tetraethylammonium bromide. The end point was detected by a color change of crystal violet indicator.

#### Infrared Absorption Spectroscopy (FTIR)

Spectra were acquired in ATR mode, with 32 scans and 4 cm^–1^ resolution using a Bruker Tensor 27 spectrometer.

#### X-ray Fluorescence (XRF)

CSBO samples were analyzed before and after extraction using XRF on a MiniPal 4 spectrometer (Panalytical), with EDS detection under a helium atmosphere. Data were processed with Omnian software based on fundamental parameters.^61^

#### Gel Content

Following ASTM D543,^62^ cylindrical specimens (1 cm Ø × 0.1 cm thick) were immersed in ethanol at 30 °C for 72 h. This solvent provides a suitable medium for dissolving amines, as well as cyclocarbonates that remain unconverted after the aminolysis step. The initial (mi) and final (mf) masses were used to calculate gel content. Three replicates were analyzed for each formulation, and standard deviation was reported.

#### Thermal Analysis and TG-IR

TGA plots were acquired on a DTA-TG STA7300 analyzer (Hitachi) from 30 to 650 °C under nitrogen flow (50 mL·min^–1^), using heating rates of 5 and 10 °C·min^–1^, the first is for better separation and identification of volatiles, and the second is to investigate the thermal behavior of PHUs. For analysis of volatile compounds, TG-IR (INVENIO S, Bruker) was employed with a transfer line heated to 150 °C. Data were processed using OriginPro Learning Edition.

## Results and Discussion

Soybean oil was epoxidized via an in situ generated peracid system composed of formic acid and hydrogen peroxide, which selectively oxidized the unsaturated bonds of the fatty acid chains to form oxirane rings. The resulting epoxidized soybean oil subsequently underwent CO_2_ insertion through ring-opening catalyzed by an alkylammonium salt, yielding soybean oil cyclocarbonate, Figure 2 (a) and (b). The obtained cyclocarbonate was employed as the matrix for the synthesis of isocyanate-free polyhydroxyurethanes (PHUs) using different polyamines and an aminoalkoxysilane as curing agents, aiming to produce hybrid materials with inorganic domains introduced by the silane phase, Figure 2 (c). All syntheses were confirmed by spectroscopic analyses, and the resulting PHUs were characterized in terms of their structural features, thermal stability, and degradation behavior, allowing a degradation mechanism to be proposed for the investigated systems.

**Figure 2:**
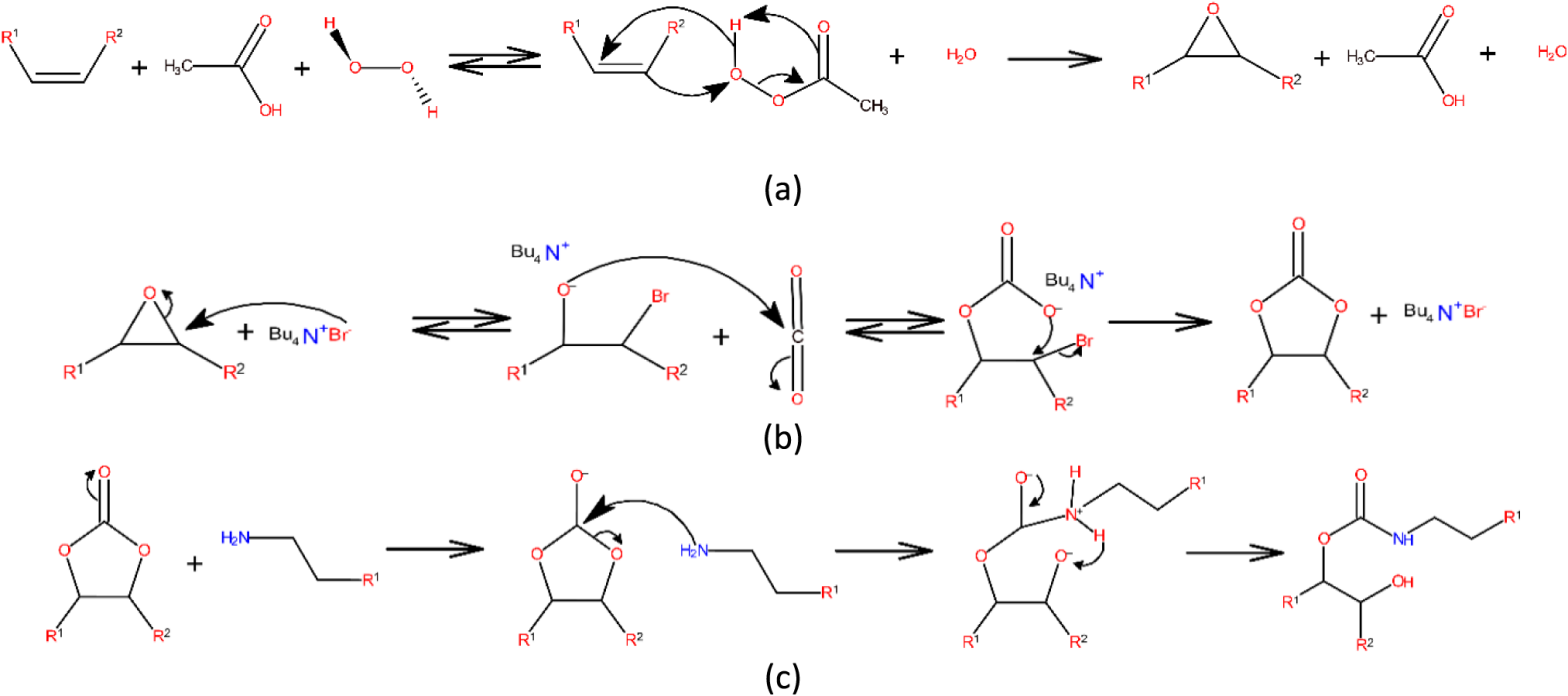
Example of reaction schemes illustrating (a) the epoxidation via the peracid route, (b) the cyclocarbonation of oxirane rings through CO_2_ capture and fixation, and (c) the aminolysis leading to hydroxyurethane formation.

### ^1^H and ^13^C Nuclear Magnetic Resonance (NMR)

Figure 3 presents the^1^ H NMR spectra of commercial soybean oil (SO, yellow line), epoxidised soybean oil (ESO, blue line), and soybean oil cyclic carbonate (CSBO, cyan line), with proton assignments consistently identified according to the lettering shown in the figure. In the SO spectrum, the signal labeled (a) at δ ≈ 5.30 ppm is assigned to the olefinic protons of the unsaturated fatty acid chains.^63^ The resonances labeled (b) at δ 4.10–4.40 ppm correspond to the methylene protons of the glycerol backbone (sn-1 and sn-3 positions). The signal labeled (c) at δ 2.75–2.80 ppm is attributed to bis-allylic protons of linoleic and linolenic units,^64,65^ while the signals labeled (d) at δ 2.00–2.30 ppm arise from allylic methylene protons adjacent to C=C bonds. The resonance labeled (e) at δ ≈ 1.60 ppm is associated with methylene protons α to the ester carbonyl groups.^66^ The intense signal labeled (f) centered at δ ≈ 1.25 ppm corresponds to the methylene groups of the long aliphatic chains, whereas the terminal methyl protons appear as the signal labeled (g) at δ 0.85–0.90 ppm.^67^

**Figure 3:**
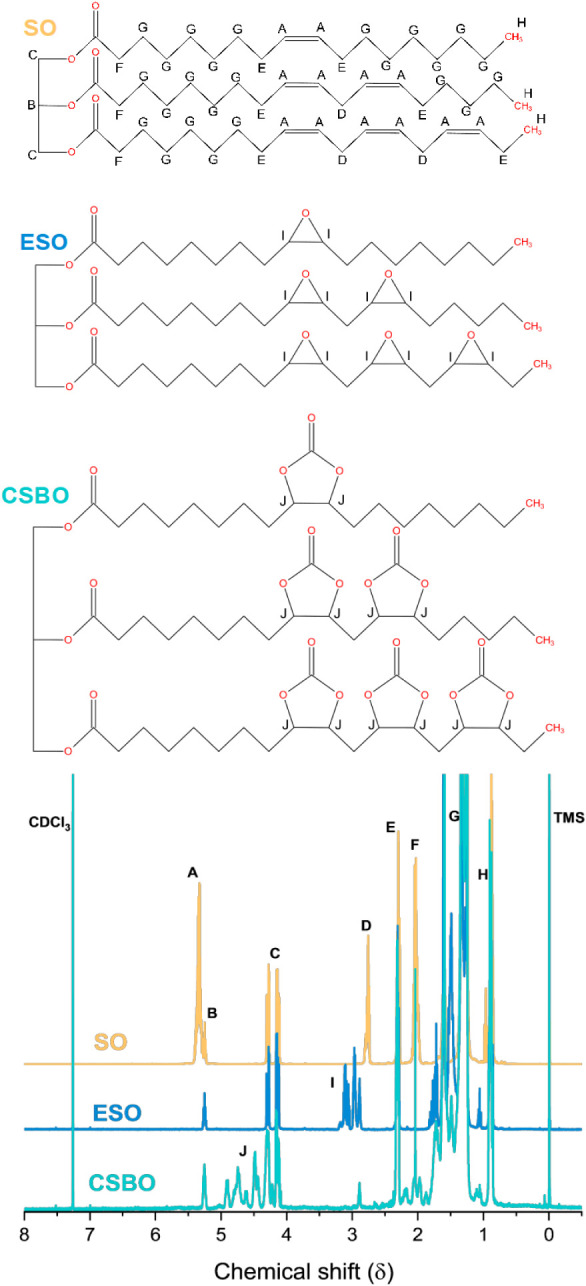
^1^H NMR spectra (400 MHz, CDCl_3_, 25 °C) of commercial soybean oil (OS, yellow line), epoxidized soybean oil (ESO, blue line), and soybean oil cyclocarbonate (CSBO, cyan line).

In the ESO spectrum, the disappearance of the olefinic signal (a) confirms the effective consumption of C=C bonds during the epoxidation step.^68^ Simultaneously, new resonances labeled **(i)** emerge in the δ 2.80–3.20 ppm region, which are attributed to protons of the oxirane rings, confirming epoxy group formation. Upon cyclocarbonation, the CSBO spectrum exhibits characteristic signals labeled **(j)** in the δ 4.40–4.90 ppm range, corresponding to methine and methylene protons adjacent to cyclic carbonate groups. The marked reduction of the epoxy-related signals **(i)** further evidence the efficient conversion of oxirane rings into cyclic carbonates.^69^ In the Supporting Information is Table S1 with the collected data from NMR spectra.

Finally, by applying the complete data set from ESO and CSBO, together with Table S1 and eqs 5, 6, and 7 from the Supporting Information, an epoxidation yield of approximately 90% was determined, even though all unsaturated bonds were consumed. This 10% discrepancy may be attributed to incomplete epoxidation, the occurrence of side reactions, or partial opening of the initially formed oxirane rings due to the presence of formic acid.^69–71^ The resulting materials exhibited an average degree of epoxidation of 4.2 epoxy groups per molecule and an epoxy-to-cyclic-carbonate conversion rate of approximately 92%, confirming the high efficiency of the synthetic procedures.

The ^13^C DEPT 135 spectra of SO, ESO, and CSBO are shown in Figure 4. In the SO spectrum, the chemical shift region between δ 120 and 135 ppm is assigned to CH carbons of the double bonds (C=C) present in the unsaturated chains. The region between δ 60 and 70 ppm corresponds to the CH and CH_2_ carbons of the glycerol backbone in triglycerides. In the δ 20–40 ppm range, a high density of signals is observed, corresponding to the CH_2_ carbons of the long aliphatic chains of fatty acids. Finally, in the δ 10–20 ppm range, a characteristic signal is identified for the terminal CH_3_ carbons of these aliphatic chains.

**Figure 4:**
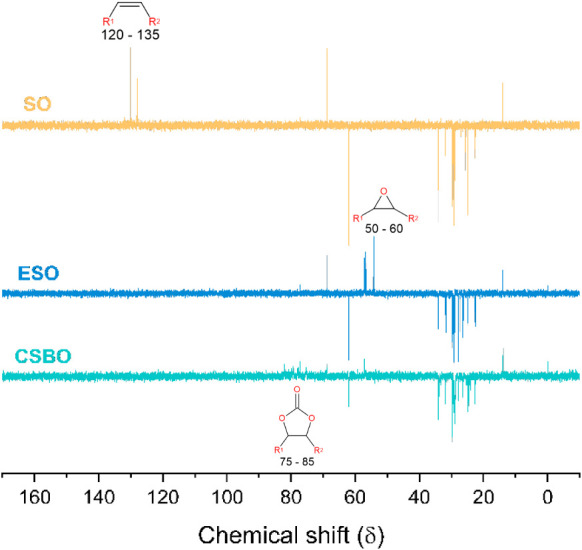
^13^C NMR spectra in DEPT 135 mode (100 MHz, CDCl_3_, 25 °C) of commercial soybean oil (OS, yellow line), epoxidized soybean oil (ESO, blue line), and soybean oil cyclocarbonate (CSBO, cyan line).

For ESO, the disappearance of the signals in the δ 120–135 ppm region, assigned to olefinic carbons of the unsaturated fatty acid chains, together with the emergence of new resonances in the δ 50–60 ppm range, confirms the successful conversion of C=C bonds into oxirane rings. The remaining chemical shifts associated with the glycerol backbone and aliphatic chains remain essentially unchanged, indicating that the epoxidation selectively affects the unsaturated sites. In CSBO, new signals appear in the δ 75–85 ppm region, which are characteristic of CH carbons adjacent to cyclic carbonate groups, clearly evidencing the formation of carbonate functionalities via CO_2_ cycloaddition. Residual signals observed in the δ 50–60 ppm region indicate the presence of unconverted oxirane rings, suggesting incomplete cyclocarbonation. The persistence of signals related to the glycerol moiety and long fatty acid chains further confirms that the backbone structure remains intact throughout the transformation sequence, in full agreement with the trends previously discussed based on the ^1^H NMR spectra.

### Titration of Epoxy Groups (ASTM D1652-04)

Titration analysis,^60^ using eqs 8, 9, and 10 from the Supporting Information, confirmed the high yields observed by NMR. The 19.5% epoxy content for the ESO indicates efficient conversion of unsaturations. The residual 1.8% epoxy content in the CSBO shows that most of the oxirane groups reacted with CO_2_, forming cyclocarbonate groups. This convergence between the methods demonstrates analytical reliability and allows the complementary use of both techniques for quantifying functionalization. Table 2 displays the acquired data.

**Table 2:** Results of Epoxy Group Titration in ESO and CSBO, According to ASTM D1652-04 Standard^a^

Material	Mass (g)	Volume (mL)	N	E (%)	W_EEW_	Yield (%)
**ESO**	0.4	15.1	0.12	19.5	220.5	-
**CSBO**	0.4	1.4	0.12	1.8	2388.8	90.7

a N = Normalization factor of the titrant solution; E = Epoxy content; W_EEW_ = Epoxy equivalent weight.

### X-ray Fluorescence (XRF)

XRF analysis (Figure 5 and Table 3) confirmed the presence of bromine (Br) from residual tetrabutylammonium bromide (TBAB) in the unpurified CSBO sample, showing a characteristic Br Kα peak at 13.28 keV. After six washing cycles with ultrapure water, this signal was significantly reduced, lowering the bromide content to below 0.3% and confirming the efficiency of the purification process.^61^ These results, consistent with the FTIR data and the qualitative AgNO_3_ test ((Figures S1 a and b)), demonstrate that the obtained material possesses high purity suitable for subsequent polymerization reactions.

**Figure 5:**
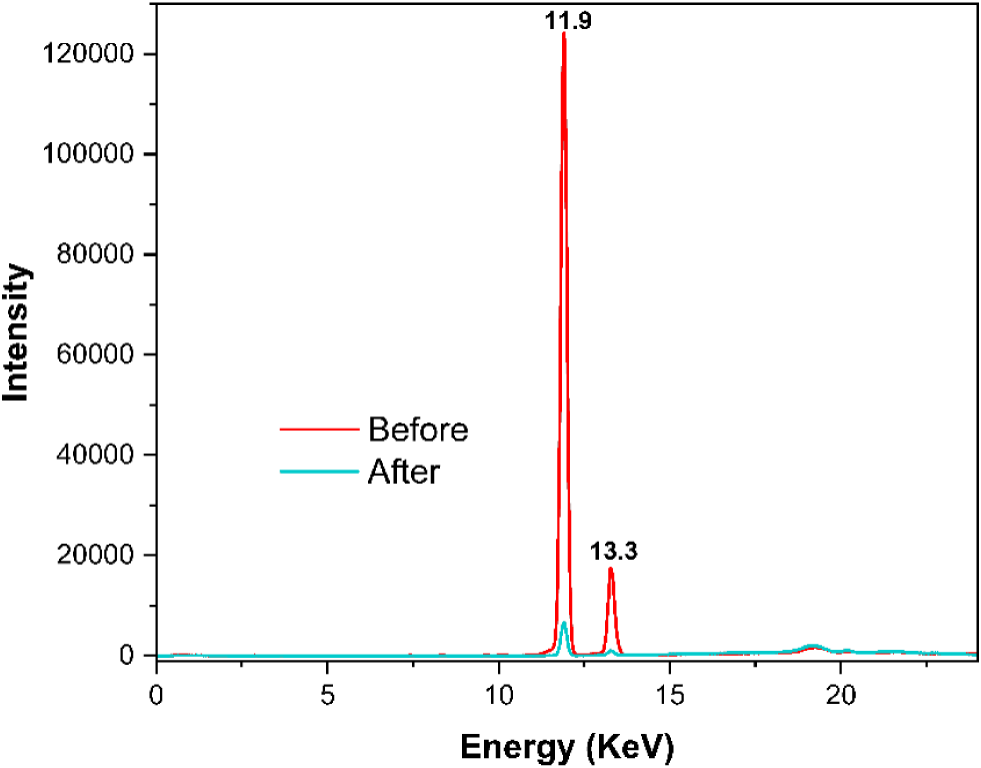
Energy-dispersive X-ray fluorescence spectra (EDS) of CSBO before (red) and after (cyan) purification through liquid–liquid extraction.

**Table 3:** XFR-EDS Data of CSBO before and after Purification Through Liquid–Liquid Extraction

		Liquid–liquid extraction	
Sample	Element	Before (%)	After (%)
**CSBO**	**Br**	10.39 ± 0.2	0.25 ± 0.01

### Fourier Transform Infrared Spectroscopy (FTIR) Analysis of Intermediates and Networks

Midinfrared spectroscopy was crucial in confirming the chemical conversions and formation of PHUs free of toxic compounds such as isocyanates and bisphenol A. The absence of the band at 3009 cm^–1^ in the ESO indicates the elimination of the SO unsaturations.^72–75^ The presence of a band at 1796 cm^–1^ in the CSBO confirms the formation of cyclic carbonate groups.^50,76^ The subsequent decrease in this band in the PHUs confirms the occurrence of aminolysis.^45,77,78^

The Amide I band at 1699 cm^–1^ and the Amide II band at 1533 cm^–1^ are attributed to the urethane bonds formed in the reaction between the amines and the cyclocarbonates.^47^ The presence of the broad band between 3100–3650 cm^–1^, characteristic of OH/NH, is also consistent with the PHU structure.^77^ It was observed that in the formulations with IPDA the band at 1796 cm^–1^ persists with greater intensity, suggesting lower conversion—possibly due to steric hindrance from the cyclic amine.^79^ Acquired spectra are presented in Figure 6, and the proposed reticulated structures for each investigated PHU are presented in Figure S2.

**Figure 6:**
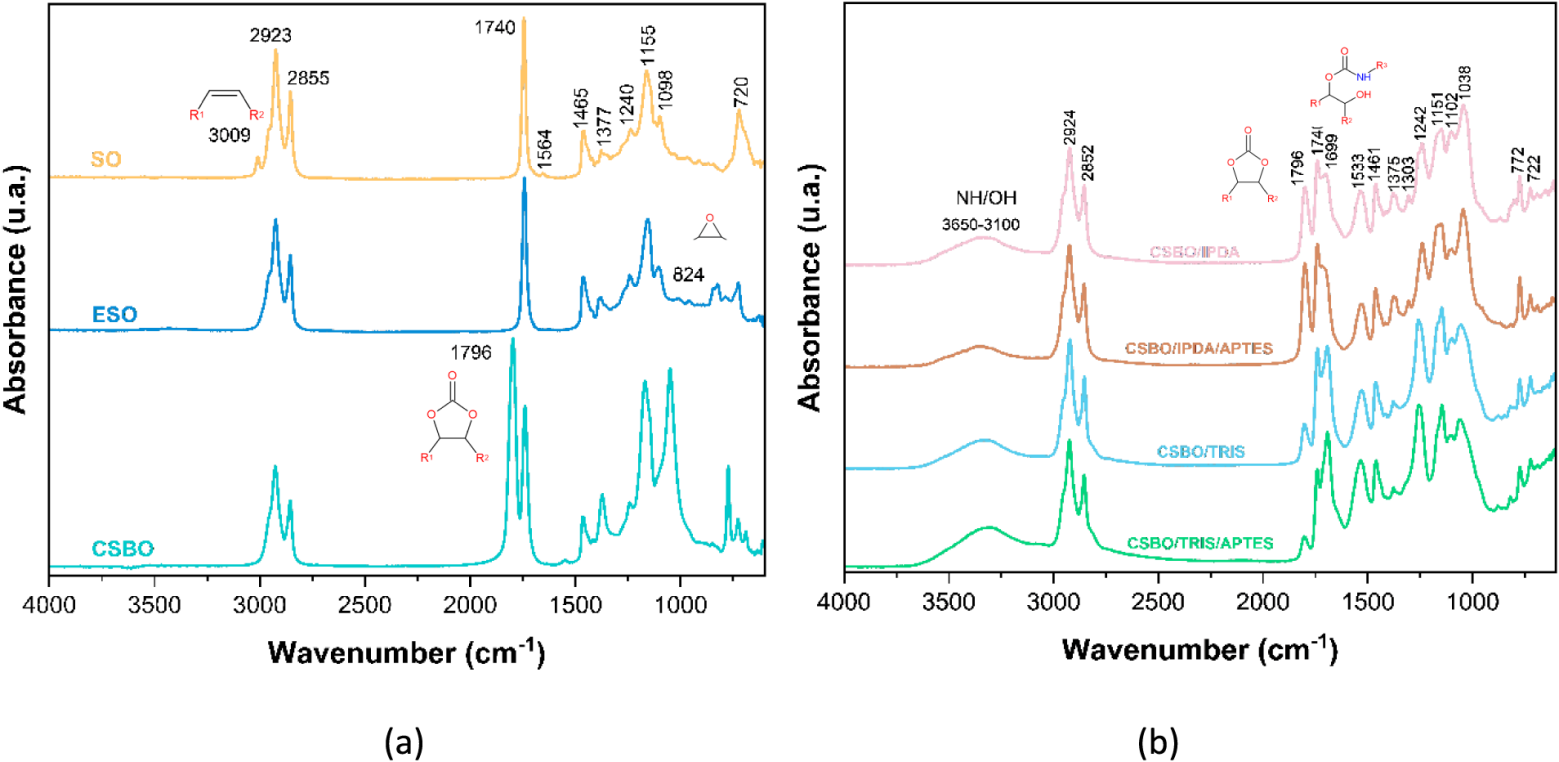
FTIR spectra. (a) SO, ESO, and CSBO. (b) Polyhydroxyurethanes CSBO/IPDA, CSBO/IPDA/APTES, CSBO/TRIS, and CSBO/TRIS/APTES.

### Gel Content—Degree of Cross-Linking and Influence of Amines

The gel content, Figure 7, determined via ethanol extraction, according to ASTM D543^63^ and provides a undirect indication of the cross-linking degree of the resulting PHUs. The results demonstrated that the amine structure strongly influences the cross-linking density.^80^ TRIS, with three primary amine groups and high conformational flexibility, yielded gel values of up to 91.9%. In contrast, IPDA, with two amine groups in a rigid structure, achieved only 71.8%.

**Figure 7:**
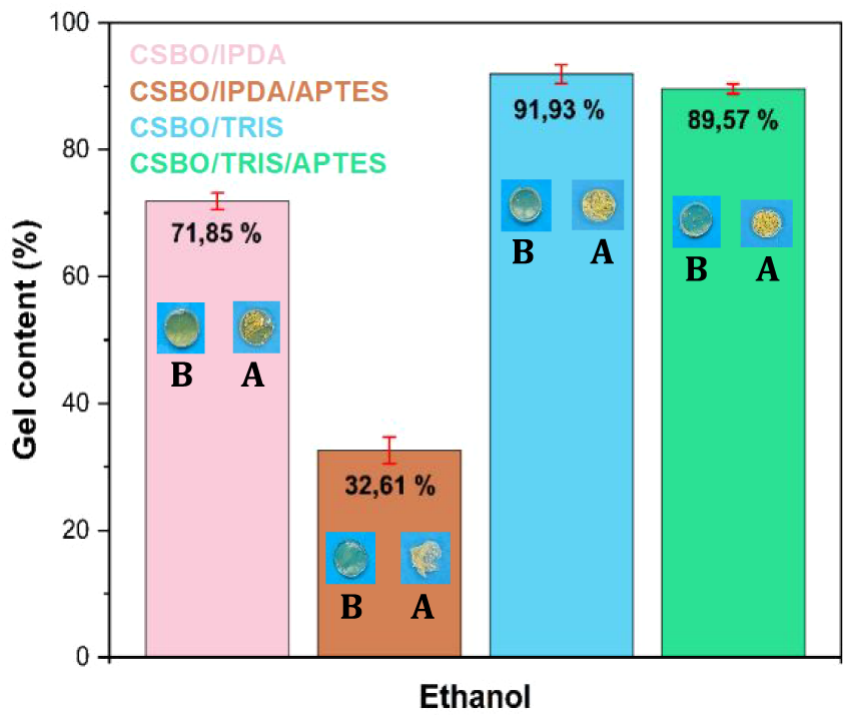
Gel content of PHUs in ethanol. Collected photos from the specimens B: before. A: after.

The presence of APTES led to distinct effects on the network formation: in formulations containing IPDA, it drastically reduced the gel content to ≈33%, whereas in those with TRIS, the reduction was only marginal. This behavior can be attributed to the hydrolysis of the ethoxy groups in APTES by the hydroxyl groups generated during aminolysis, leading to the formation of siloxane domains that are inert toward urethane bond formation.^47–49,81,82^ These findings demonstrate that the chemical structure and functionality of the amine play a critical role in network development, with TRIS enabling a more robust and cross-linked system even in hybrid formulations.

### Thermal Stability and Degradation Behavior

Figure 8 presents the TGA and DTG plots acquired a heating rate of 10 °C·min^–1^ (a) and comparison of gel content with thermal degradation (b) for the investigated formulations. Table 4 summarizes the thermal degradation parameters derived from the TGA curves.

**Figure 8:**
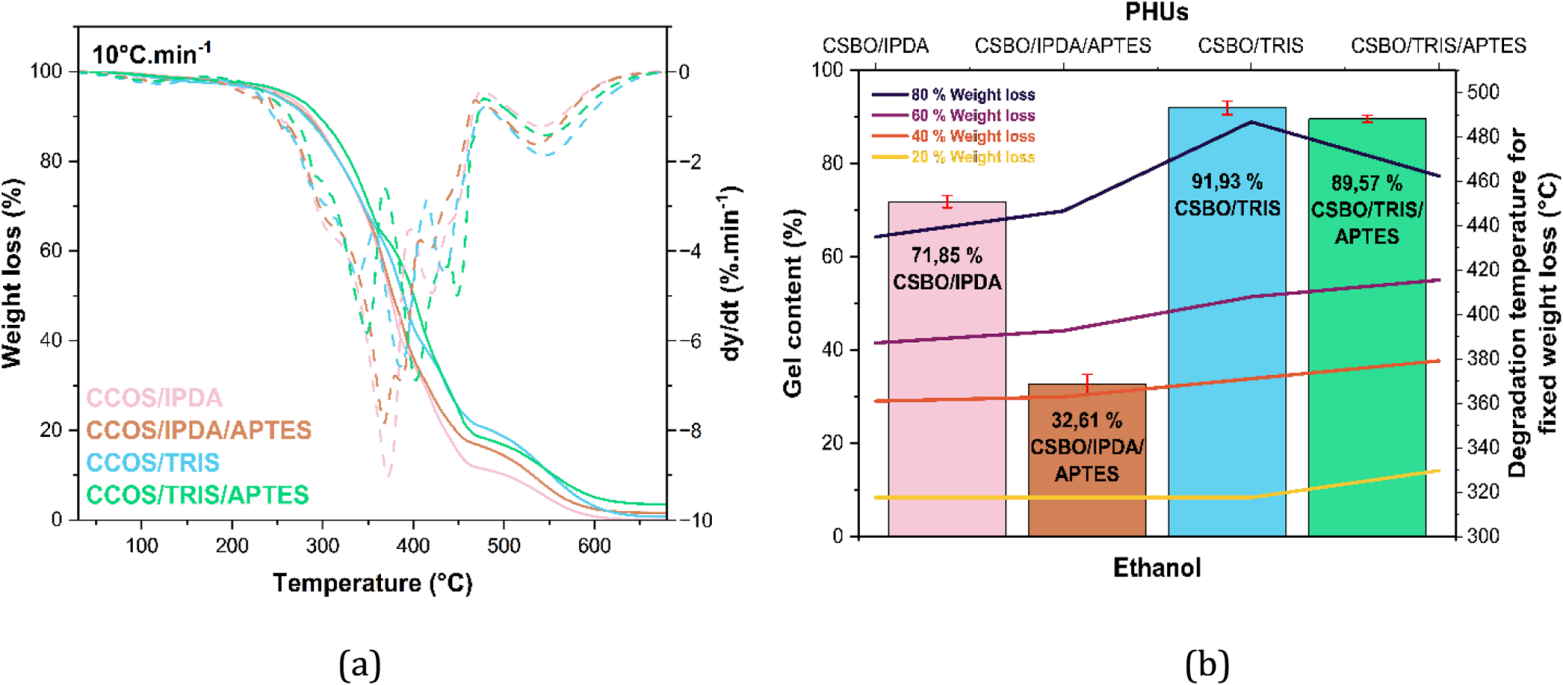
TGA and DTG plots acquired at 10 °C.min^–1^ (a) and comparison of gel content with thermal degradation (b) for the indicated formulations.

**Table 4:** Thermogravimetric Parameters of the PHUs Collected at 10 °C.min^–1^ ^a^

		Temperature (°C)				D_max_ (%.min^–1^)		
			T_p_					
Event	Formulation	T_i_	T_p1_	T_p2_	T_f_	D_max1_	D_max2_	Mass loss (%)
**Water loss**	**CSBO/IPDA**	50	119	-	170	0.2	-	1.6
	**CSBO/IPDA/APTES**	50	110	-	170	0.2	-	1.4
	**CSBO/TRIS**	50	118	-	170	0.3	-	2.5
	**CSBO/TRIS/APTES**	50	111	-	170	0.2	-	1.8
**Free primary amine degradation**	**CSBO/IPDA**	175	224	-	250	1.1	-	2.9
	**CSBO/IPDA/APTES**	175	230	-	250	1.4	-	3.6
	**CSBO/TRIS**	175	235	-	250	1.2	-	1.5
	**CSBO/TRIS/APTES**	175	240	-	250	1.2	-	1.8
**Degradation with low degree of cross-linked of PHUs**	**CSBO/IPDA**	250	371	-	396	8.9	-	58.7
	**CSBO/IPDA/APTES**	250	368	-	406	7.8	-	61.9
	**CSBO/TRIS**	250	335	-	356	4.5	-	30.4
	**CSBO/TRIS/APTES**	250	346	-	367	5.8	-	32.6
**Degradation with high degree of cross-linked of PHUs**	**CSBO/IPDA**	398	417	444	472	4.9	3.2	26.1
	**CSBO/IPDA/APTES**	408	418	446	468	3.9	2.6	15.9
	**CSBO/TRIS**	360	385	435	473	6.6	4.4	44.8
	**CSBO/TRIS/APTES**	368	402	454	474	6.8	4.9	45.4
**Carbonization of degradation products**	**CSBO/IPDA**	477	540	-	650	1.2	-	10.7
	**CSBO/IPDA/APTES**	473	534	-	650	1.6	-	15.5
	**CSBO/TRIS**	475	548	-	650	1.8	-	20.8
	**CSBO/TRIS/APTES**	476	548	-	650	1.4	-	14.9

a T_i_, T_p_, and T_f_: Initial, peak, and final temperatures of each event. d_max_: Maximum degradation rate at each stage.

All PHU networks exhibited comparable multistep degradation profiles. Based on the bond enthalpies within the investigated PHU networks allows the identification of structural sites most prone to dissociation, providing insight into the degradation pathways at each stage.^83,84^ The initial mass loss (50–170 °C) is attributed to the evaporation of residual water, followed by a second stage (175–250 °C), corresponding to the decomposition of unreacted amines. The principal degradation region (250–475 °C) is associated with scission of the polymer network, where systems with lower cross-link density underwent degradation at earlier stages.

PHUs cured with IPDA predominantly degraded between ≈250–400 °C, indicating limited cross-linking due to the steric hindrance of the cycloaliphatic diamine. In contrast, TRIS-based networks exhibited two distinct degradation domains: a lower-temperature phase (≈250–360 °C) and a high-temperature phase (≈365–475 °C), reflecting the formation of highly cross-linked segments promoted by the trifunctional nature of TRIS. The final degradation stage (≈475–650 °C) is assigned to the carbonization of residual material.

Overall, the incorporation of APTES tended to reduce the cross-link density of the PHU networks, particularly when compared with systems cured exclusively with IPDA or TRIS, which contain two and three primary amines, respectively. The proposed mechanism involves the hydrolysis of the ethoxy groups of APTES, followed by the condensation of the resulting silanol groups, which generates water simultaneously with the aminolysis of the carbonate units. This process leads to the formation of siloxane domains that are inert toward urethane bond formation. Incomplete hydrolysis of the silanol groups, resulting in dangling chains as end groups, together with the plasticization effect of the generated water, could contribute to the observed decrease in cross-linking density upon incorporation of APTES, as also corroborated by the gel content results.

The obtained data are consistent with those reported in the literature. Poussard et al. (2016)^50^ produced PHUs from soybean oil using different diamines and observed thermal stability up to 200 °C, attributing the initial mass loss event to the degradation of the employed diamines. Similarly, Morales-González et al. (2024)^38^ investigated various PHU formulations from soybean oil with diamines and also observed thermal stability around 200 °C, they attributed the mass loss observed between 50 and 100 °C to moisture absorbed by the samples and reported that the subsequent thermal events correspond to the degradation of urethane bonds, followed by degradation of the polymer matrix.

According to Figure 8 (b), it is evident that the higher the gel content, the greater the thermal stability of the PHU. This is demonstrated by the shift of the degradation temperatures to higher values at fixed mass loss percentages, a behavior directly associated with the higher cross-link density, which restricts chain mobility and delays macromolecular scission.^84^

### DTG Curve Deconvolution

To better resolve the overlapping thermal events observed in the DTG profiles of the PHUs, a Gaussian deconvolution was applied, and the data are presented in Figure 9. Equation 11, Figure S3 and Table S2 in the Supporting Information summarize the adopted methodology. This approach enabled the identification of distinct degradation stages and provided insights into the structural characteristics of each formulation.

**Figure 9:**
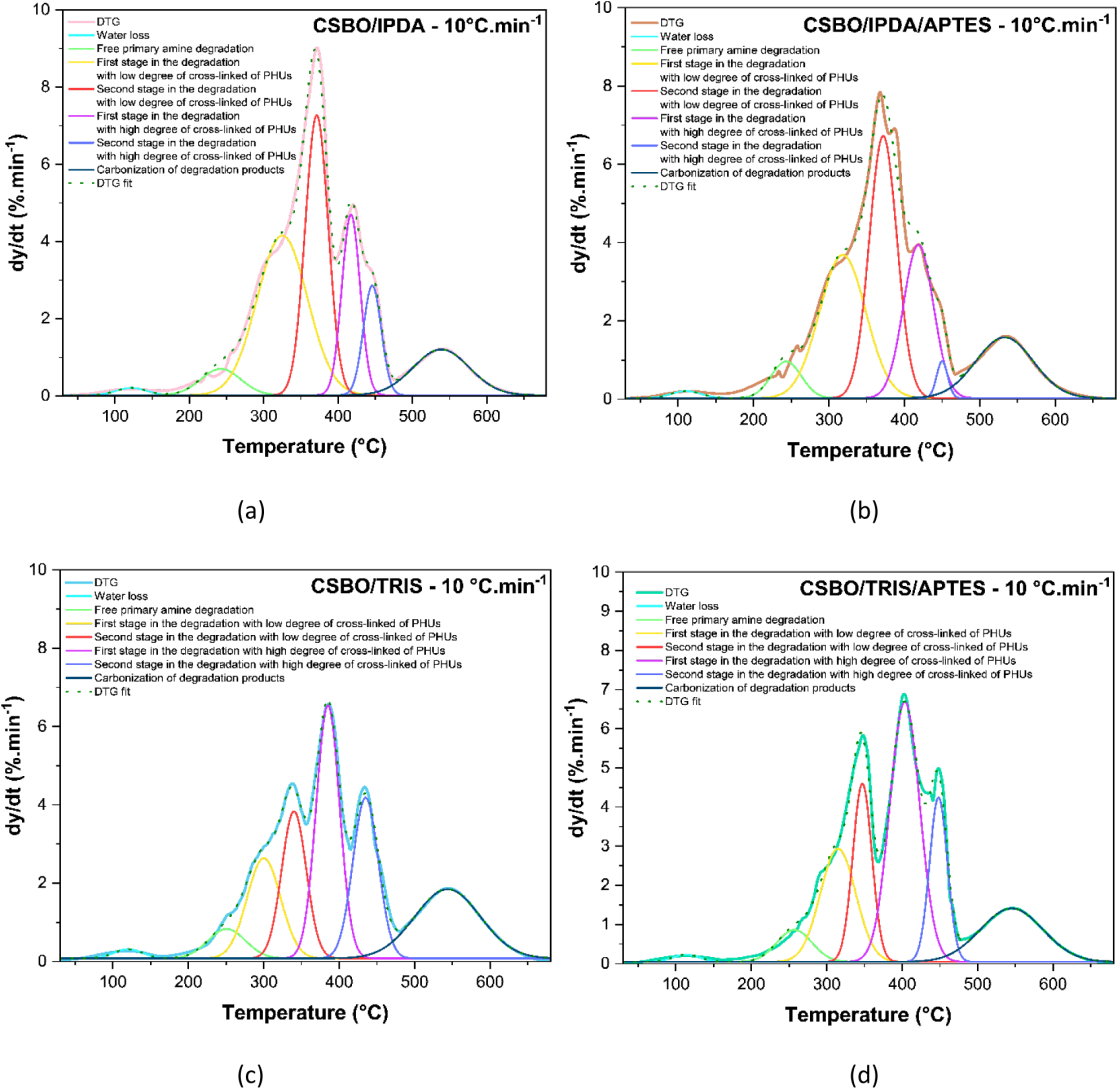
Deconvolution of DTG at the rate of 10 °C·min^–1^ for CSBO/IPDA (a), CSBO/IPDA/APTES (b), CSBO/TRIS (c), and CSBO/TRIS/APTES (d).

The deconvoluted DTG curves revealed four main degradation events across all PHU networks, and the dissociation at each stage was performed based on the binding energy of each band:^83,84^

●Event I (≈50–170 °C): attributed to the release of residual water from the sol–gel process during aminolysis, cyan peak;

●Event II (≈190–230 °C): attributed to the degradation of unreacted or loosely bound amines, green peak;

●Event III (≈250–360 °C): corresponding to the decomposition of low-density cross-linked segments within the polymer matrix, yellow and red peaks;

●Event IV (≈365–475 °C): related to the breakdown of densely cross-linked domains, particularly pronounced in TRIS-based formulations, pink and blue peaks;

●Event V (>475 °C):associated with advanced carbonization processes and the formation of thermally stable residues, black peak.

The relative intensity and area under each deconvoluted peak provided quantitative support for the influence of the curing agent structure on the degradation pathway.^84^ TRIS-based PHUs exhibited dominant contributions from Event IV, consistent with the presence of highly cross-linked networks. Conversely, IPDA- and APTES-containing systems showed greater contributions from Event III, indicating a prevalence of less stable and loosely reticulated regions.

These findings align with the gel content and TGA data, further reinforcing the correlation between amine structure, network architecture, and thermal degradation behavior. The use of DTG deconvolution thus proves essential for unraveling complex degradation mechanisms in multifunctional PHU systems.

### TG-IR Analysis of Soybean-Based PHUs

Infrared spectroscopy coupled with thermogravimetric analysis (TG-IR) enabled the identification of the main thermal degradation mechanisms of soybean oil-based PHUs (poly (hydroxyurethanes)), synthesized using the amines IPDA, TRIS, and the silane APTES. The main volatiles detected included water, amines, amides, hydrocarbons, carbon dioxide, ketenes, and others. From the 2D spectra and quantitative peak area data, the following mechanisms were identified and discussed. Collected data are presented in Figures 10, 11 and Table 5.

**Figure 10:**
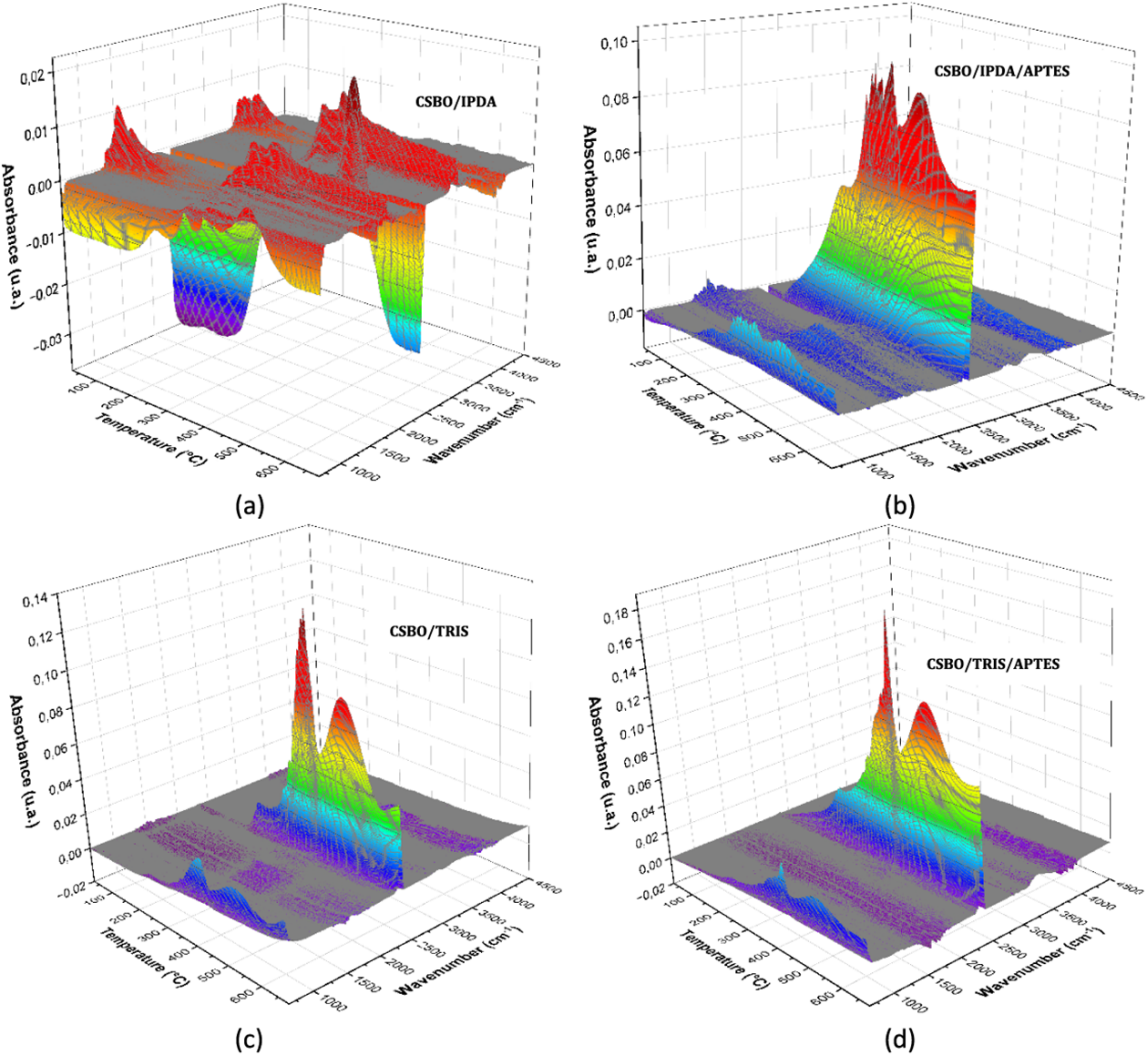
3D infrared spectra of volatile compounds at a heating rate of 5 °C·min^–1^. CSBO/IPDA (a), CSBO/IPDA/APTES (b), CSBO/TRIS (c), and CSBO/TRIS/APTES (d).

**Figure 11:**
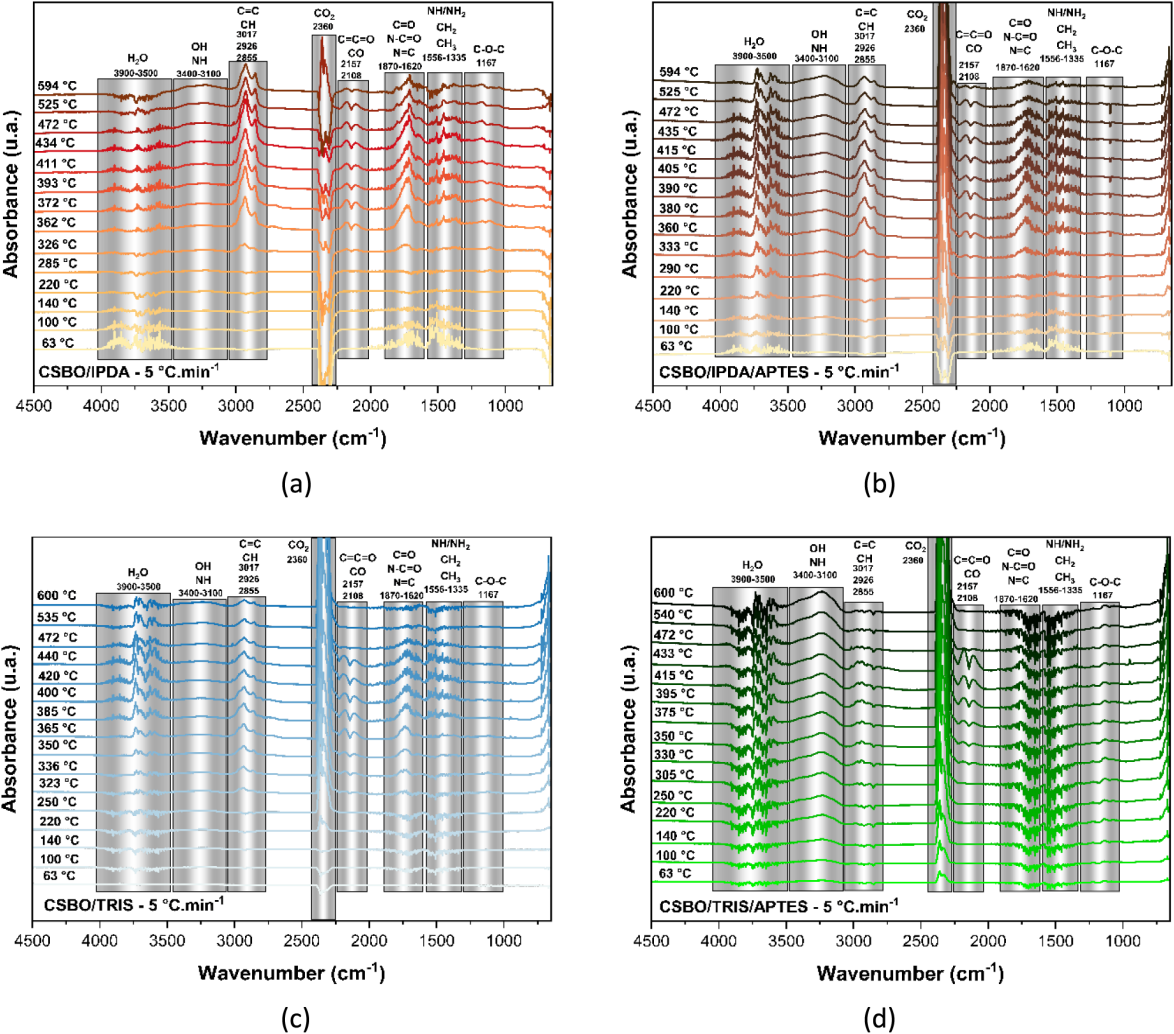
2D infrared spectra of volatile compounds. CSBO/IPDA (a), CSBO/IPDA/APTES (b), CSBO/TRIS (c), and CSBO/TRIS/APTES (d), degradation temperatures indicated.

**Table 5:** FTIR Peaks Area of PHUs’ Volatile Compounds Collected from TG-IR^a^

		**Area in the wavenumber** **(cm** ^**–1**^ **)** **range**							
Temperature (°C)	Formulation	3900–3500	3400–3100	3017–2855	2360	2157–2108	1870–1620	1556–1335	1167
63	**CSBO/IPDA**	0.66	-	-	-	-	0.47	0.58	-
	**CSBO/IPDA/APTES**	0.36	-	-	-	-	0.23	0.29	-
	**CSBO/TRIS**	-	-	-	-	-	-	-	-
	**CSBO/TRIS/APTES**	-	-	-	-	-	-	-	-
220	**CSBO/IPDA**	-	0.05	-	-	-	0.06	0.03	-
	**CSBO/IPDA/APTES**	0.17	0.15	-	0.84	0.01	0.05	0.08	-
	**CSBO/TRIS**	-	0.03	-	0.17	-	-	-	-
	**CSBO/TRIS/APTES**	-	0.31	-	1.13	0.03	-	-	0.03
≈330	**CSBO/IPDA**	0.10	0.08	0.19	-	0.06	0.21	0.12	0.02
	**CSBO/IPDA/APTES**	0.33	0.28	0.17	2.18	0.03	0.26	0.16	0.02
	**CSBO/TRIS**	0.16	0.08	0.12	1.13	0.04	0.18	0.07	0.01
	**CSBO/TRIS/APTES**	0.28	0.45	0.11	2.77	0.05	0.21	0.14	0.03
≈430	**CSBO/IPDA**	0.22	0.14	1.07	-	0.23	0.73	0.27	0.05
	**CSBO/IPDA/APTES**	0.73	0.30	0.48	4.22	0.15	0.59	0.42	-
	**CSBO/TRIS**	0.62	0.09	0.31	6.23	0.15	0.45	0.27	-
	**CSBO/TRIS/APTES**	0.70	0.56	0.23	6.98	0.39	0.34	0.28	-
≈600	**CSBO/IPDA**	-	0.31	0.60	-	0.09	0.41	0.13	0.03
	**CSBO/IPDA/APTES**	0.28	0.49	0.26	2.88	0.07	0.23	0.09	-
	**CSBO/TRIS**	0.27	0.12	0.15	3.07	-	0.17	0.18	-
	**CSBO/TRIS/APTES**	0.39	0.91	0.10	5.08	-	-	-	-

a Thepeaks were integrated without curve normalization, using the Bruker OPUS Software Version 8.5 through the interactive method with integration mode B.

●Thermal hydrolysis:^85^ broad bands between 3900–3500 cm^–1^, attributed to the presence of – OH groups (from water and silanols), were detected from as early as 63 °C. These bands increased progressively until advanced degradation (∼430 °C), indicating urethane cleavage, macromolecule cleavage, and decomposition of silanol groups formed with APTES. The marked increase of these bands in the APTES-containing formulations suggests higher moisture retention, likely due to the formation of hydrophilic siloxane structures.

●Cleavage of urethane and amide bonds:^86^ bands in the region of 1870–1355 cm^–1^, observed from low temperatures, indicate the decomposition of N-substituted amides and urethanes, suggesting the onset of degradation of the non-cross-linked polymer matrix. The sharp increase in these bands from 250 °C to 430 °C, particularly for IPDA, is associated with the cleavage of NH–COO and NH–CO–NH bonds.

●Formation of amines, ketenes, CO_2_, and hydrocarbons:^87,88^ bands between 2157–2108 cm^–1^ (ketenes/nitriles) and 2360 cm^–1^ (CO_2_) appeared at intermediate temperatures (∼330–430 °C), suggesting decarboxylation reactions and the breakdown of ureas or cyclic carbonates. Bands between 3017–2855 cm^–1^ indicate the formation of hydrocarbons, possibly due to the cleavage of aliphatic chains from triglycerides.

●Interactions with silanols (APTES):^82^ APTES significantly influenced the thermal mechanisms. In APTES-based formulations, −OH bands (3400–3100 cm^–1^) were intensified, and the areas of the CO_2_ and NH/–OH bands increased. This indicates that, in addition to acting as a nucleophile in the opening of cyclic carbonates, APTES promotes additional cross-linking through silanol–silanol condensation (Si–OH ⇌ Si–O–Si + H_2_O), forming a hybrid organic–inorganic network.

●Structural effects of amines:

•IPDA (rigid cyclopentyl diamine): showed lower reactivity (likely due to steric hindrance), as evidenced by intense bands of unreacted amines and amides at temperatures below 250 °C, suggesting a less cross-linked network.

•TRIS (triamine): the presence of three primary amine groups promotes a higher degree of cross-linking, reflected in lower volatile release during early thermal stages. TG-IR data showed that TRIS-based formulations exhibited lower peak areas up to 250 °C, confirming a higher conversion rate during synthesis.

•APTES: induced increased release of hydroxylated volatiles and intensified peaks for CO_2_ and ketenes, suggesting the formation of a hybrid network with distinct thermal and dynamic characteristics. The formation of Si–O–Si contributes to thermal stability and potential adaptability of the network.

### Proposed Thermal Degradation Mechanism of Soybean-Based PHUs

Building upon the thermal and spectroscopic analyses, particularly the TG-IR and deconvoluted DTG data, a general degradation mechanism for soybean oil-derived PHUs is proposed, as illustrated in Figure 11. This mechanism accounts for the main bond types present in the PHU structure and considers their respective bond dissociation enthalpies (Table 6), supporting the identification of fragments observed in the evolved gases.

**Table 6:** Bond Enthalpies of Produced PHUs’ Molecules from Soybean Oil^a^

Bond type	Bond enthalpy (kJ·mol^–1^)
**H–N**	393
**H–O**	460
**C–H**	414
**C–C**	347
**C–N**	276
**C–O**	351
**C=O**	745

a Adapted from Chang, R. and Goldsby, K. A. (2013).^83^

The degradation pathway can be divided into two principal domains:

●Urethane phase degradation: this phase involves cleavage of urethane linkages, leading to multiple degradation products depending on the network environment:

•Dissociation into isocyanates and alcohols: typically associated with retro-urethane reactions, although in PHUs (nonisocyanate), this may represent theoretical reversibility.

•Formation of secondary or primary amines and olefins: evidenced by TG-IR bands in the region of 2157–2108 cm^–1^ (ketenes/olefins), particularly under high-temperature conditions.

•Reversible scission into cyclic carbonate and primary amines: aligned with the synthetic route, suggesting the potential regeneration of starting materials under specific conditions that require further study.

●Triglyceride phase degradation: this phase involves the breakdown of the residual fatty acid backbone, consistent with the presence of aliphatic and carboxylic fragments in evolved gases:

•Decarboxylation reactions, leading to the release of CO_2_ (2360 cm^–1^).

•C–C and C–H bond scissions, resulting in hydrocarbons and glycerol derivatives, such as ketenes.

•Glycerol cleavage pathways, supported by the detection of water and hydroxyl-rich volatiles in TG-IR spectra.

Table 6 summarizes bond dissociation enthalpies of relevant functional groups, which support the temperature ranges at which major degradation events occur, as observed experimentally.

These values support the hypothesis that ester, hydrocarbon, carbonyl, and amine groups were generated during the scission of the macromolecule, as observed in Events III and IV of the DTG deconvolution (≈250–475 °C) and in the spectra of the gases released in the TG-IR (≈250–600 °C). Figure 12 illustrates the proposed degradation mechanism based on the experimental data and literature precedent.^56–59^

**Figure 12:**
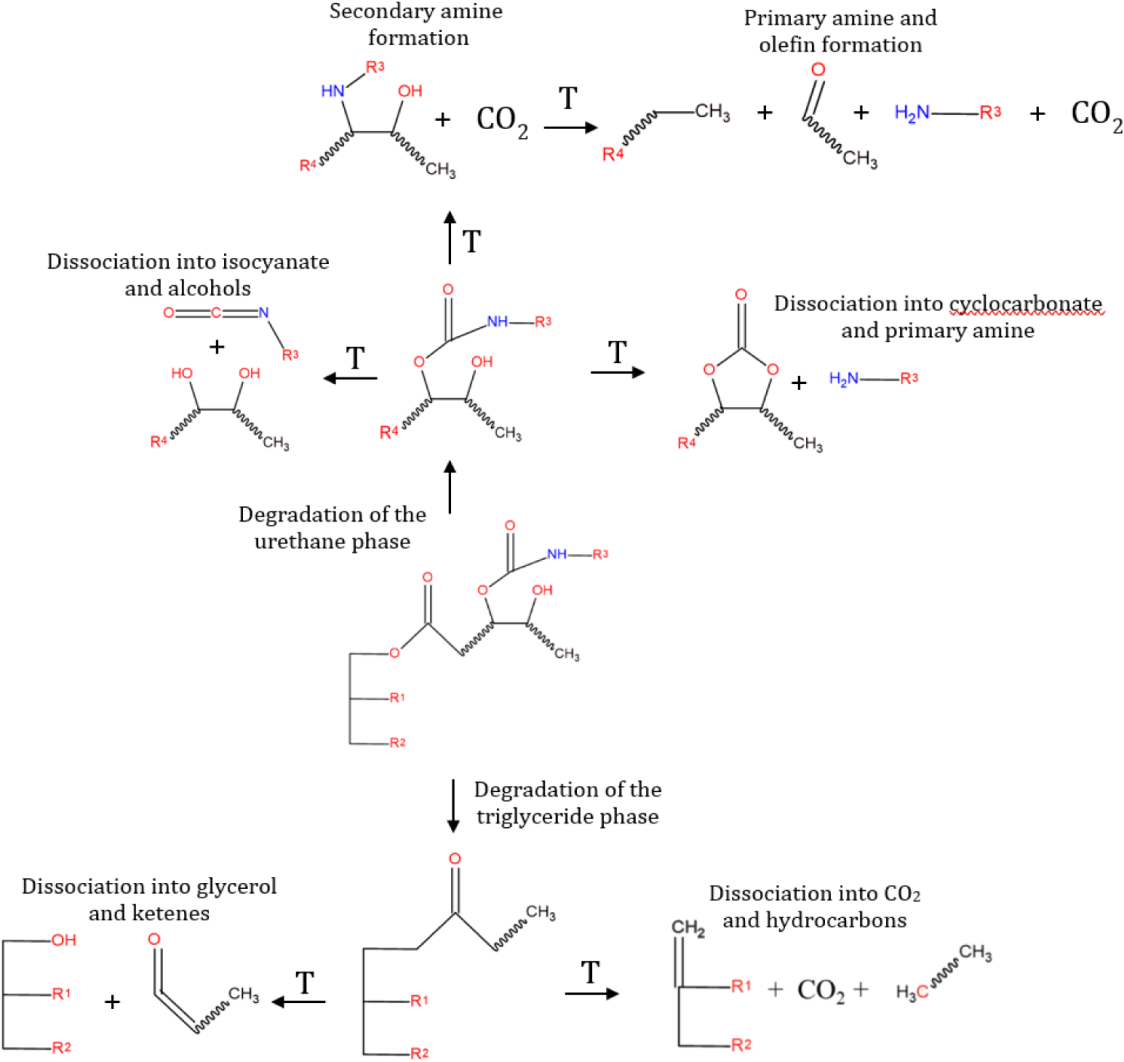
Proposed degradation mechanism for soybean oil-based PHUs.

## Main Achievements

●**Scientific:** this study provides an unprecedented and in-depth characterization of the thermal degradation mechanisms of soybean oil-derived PHUs, combining TG-IR spectroscopy, thermogravimetric analysis, and peak deconvolution to identify key volatiles and degradation pathways. The experimental evidence highlights the structural influence of the amines (TRIS, IPDA) and the silane APTES on the stability and architecture of the polymer networks, contributing to the understanding of structure–property–thermal behavior correlations in nonisocyanate polyurethane systems.

●**Technological:** a clean and efficient synthetic route was successfully established for the production of soybean oil-based PHUs, involving sequential epoxidation, CO_2_ cycloaddition (≈92% yield), and aminolysis under mild conditions (T < 120 °C, P ≈ 1.3 MPa) using TBAB as catalyst. These networks may exhibit distinct cross-linking densities depending on the amine employed, with TRIS-based formulations reaching up to 91.9% gel content. Incorporation of APTES enabled the formation of hybrid organic–inorganic domains. Moreover, spectroscopic and thermal analyses (TG-IR, FTIR, TGA) revealed dynamic covalent motifs such as urethane, urea, and silanol groups, along with ketene formation, suggesting that these networks exhibit structural features compatible with vitrimeric behavior.

●**Environmental:** the work marks a significant environmental milestone by fully replacing toxic diisocyanates with a safe route based on renewable feedstocks and CO_2_ chemical fixation, thus contributing to impact reduction and aligning with green chemistry principles and the United Nations Sustainable Development Goals (SDGs 9, 12, and 13).

●**Economic:** the use of soybean oil—a widely available, low-cost, and locally sourced raw material—combined with the feasibility of synthesis under mild conditions and with standard equipment, enables the scalable production of thermally stable polymers that are competitive in the biobased materials market.

●**Social:** by eliminating the use of diisocyanates, this work enhances occupational safety and public health, while promoting responsible innovation and interdisciplinary training. It supports the development of highly qualified professionals in the fields of green chemistry and biobased materials engineering.

## Conclusion

This study demonstrated a successful approach for the synthesis of poly­(hydroxyurethanes) (PHUs) derived from soybean oil via epoxidation, CO_2_ cycloaddition and aminolysis, using accessible reagents and mild processing conditions. The resulting PHUs exhibited distinct network architectures depending on the chemical structure of the polyamines used—particularly highlighting the effect of TRIS in promoting highly cross-linked networks (up to 91.9% gel content), and the influence of APTES in introducing hybrid organic–inorganic domains, as confirmed by FTIR.

Thermogravimetric analysis (TGA) coupled with evolved gas infrared spectroscopy (TG-IR) and DTG deconvolution revealed multistep thermal degradation processes, with variations directly associated with the degree of cross-linking and presence of siloxane structures. The main volatiles identified—CO_2_, amines, ketenes, and water—supported a proposed degradation mechanism that involves cleavage of urethane and amide bonds, retro-aminolysis, and decomposition of triglyceride backbones.

Importantly, the detection of dynamic functional motifs (NH–CO–NH, NH–CO–O, Si–OH) and ketene intermediates suggests reprocessing adaptability in the PHU networks, particularly in formulations containing TRIS and APTES. These findings support the hypothesis that such networks may exhibit dynamic covalent rearrangements under appropriate thermal or humidity conditions, although further validation through rheological and recyclability tests is warranted.

Overall, this work contributes valuable insight into the design, structure–property relationships, and thermal degradation behavior of nonisocyanate polyurethanes, reinforcing their viability as safer, renewable and functionally versatile alternatives to conventional PUs. The outcomes also align with international priorities for biobased polymers development and green chemistry, offering technological, environmental, and occupational health benefits for future applications in advanced materials engineering.

## Supplementary Material



## Data Availability

The data supporting this study are available within the manuscript.

## Notes

Additional experimental details and quantitative analyses supporting the structural and thermal characterization are provided in the Supporting Information. These include: (i) the complete assignment of the main ^1^H NMR signals of soybean oil (SO), epoxidised soybean oil (ESO), and soybean oil cyclic carbonate (CSBO), together with the equations used to calculate the molar mass and iodine value from ^1^H NMR data; (ii) the estimation of epoxidation and cyclocarbonation yields based on combined ^1^H NMR integration and epoxy titration analyses (Table S1 and Equations S1–S10); (iii) qualitative monitoring of catalyst removal by AgNO_3_ tests during liquid–liquid extraction (Figure S1); (iv) proposed molecular network structures for all PHU formulations (Figure S2); and (v) the Gaussian deconvolution methodology applied to DTG curves, including fitting equations, peak parameters, and correlation coefficients (Figure S3 and Table S2). These supplementary data ensure full transparency of the analytical procedures and provide the quantitative basis for the discussion of conversion efficiency, network architecture, and thermal degradation mechanisms presented in this work (PDF)

## References

[1] de
Lima A. L., Domingos Gonçalves A., Rodrigues
Fernandes D., Custódio dos Santos T. J. A., Mota C. (2024). Química
e Circularidade. Rev. Virtual Quím..

[2] Nikolaivits E., Pantelic B., Azeem M., Taxeidis G., Babu R., Topakas E., Brennan
Fournet M., Nikodinovic-Runic J. (2021). Progressing
Plastics Circularity: A Review of Mechano-Biocatalytic Approaches
for Waste Plastic (Re)­Valorization. Front. Bioeng.
Biotechnol..

[3] Ecochard Y., Caillol S. (2020). Hybrid Polyhydroxyurethanes:
How to Overcome Limitations
and Reach Cutting Edge Properties?. Eur. Polym.
J..

[4] Centeno-Pedrazo A., Perez-Arce J., Freixa Z., Ortiz P., Garcia-Suarez E. J. (2024). Non-Isocyanate
Polyurethanes Derived from Carbonated Soybean Oil: Synthesis, Characterization
and Comparison with Traditional Vegetable Oil-Based Polyurethanes. Prog. Org. Coat..

[5] Gomollón-Bel F. (2019). Ten Chemical
Innovations That Will Change Our World: IUPAC Identifies Emerging
Technologies in Chemistry with Potential to Make Our Planet More Sustainable. Chem. Int..

[6] Gomollón-Bel F., García-Martínez J. (2023). Chemical Solutions to the Current
Polycrisis. Angew. Chem., Int. Ed..

[7] United Nations THE 17 GOALS | Department of Economic and Social Affairs - Sustainable Development. https://sdgs.un.org/goals. (accessed 2025–March–24).

[8] Bayer O. (1947). Das Di-Isocyanat-Polyadditionsverfahren
(Polyurethane). Angew. Chem..

[9] Sharmin, E. ; Zafar, F. Polyurethane: An Introduction. In Polyurethane; InTechOpen, 2012. DOI: 10.5772/51663.

[10] Polaris Market Research. Polyurethane (PU) Market Size & Share Global Analysis Report, 2024–2032. https://www.polarismarketresearch.com/industry-analysis/polyurethane-pu-market. (accessed 2025–March–24).

[11] Fortune Business Insights. Polyurethane (PU) Market Size & Share Report, 2024–2032. https://www.fortunebusinessinsights.com/industry-reports/polyurethane-pu-market-101801. (accessed 2025–March–24).

[12] Fact.MR Polyurethane Market Report, 2024–2032. https://www.factmr.com/report/polyurethane-market. (accessed 2025–March–24).

[13] Grand View Research Polyurethane (PU) Market Size, Share & Trends Analysis Report, 2024–2032. https://www.grandviewresearch.com/industry-analysis/polyurethane-pu-market. (accessed 2025–March–24).

[14] Precedence Research Polyurethane Market Size, Share & Trends Analysis Report, 2024–2032. https://www.precedenceresearch.com/polyurethane-market. (accessed 2025–March–24).

[15] Mordor Intelligence Polyurethane Market - Growth, Trends, And Forecast (2024–2032). https://www.mordorintelligence.com/industry-reports/polyurethane-market. (accessed 2025–March–24).

[16] Rüegger M., Droste D., Hofmann M., Jost M., Miedinger D. (2014). Diisocyanate-Induced
Asthma in Switzerland: Long-Term Course and Patients’ Self-Assessment
after a 12-Year Follow-Up. J. Occup Med. Toxicol..

[17] Ṡwierczyńska-Machura D., Brzeźnicki S., Nowakowska-Ṡwirta E., Walusiak-Skorupa J., Wittczak T., Dudek W., Bonczarowska M., Wesolowski W., Czerczak S., Pałczyński C. (2015). Occupational
Exposure to Diisocyanates in Polyurethane Foam Factory Workers. Int. J. Occup. Med. Environ. Health.

[18] European Union Commission Regulation (EU) 2020/1149 of 3 August 2020 amending Annex XVII to Regulation (EC) No 1907/2006 of the European Parliament and of the Council concerning the Registration, Evaluation, Authorisation and Restriction of Chemicals (REACH) as regards; Official Journal of the European Union. https://eur-lex.europa.eu/legal-content/EN/TXT/?uri=CELEX%3A32020R1149. (accessed 2025–March–25).

[19] Figovsky O., Shapovalov L., Axenov O. (2004). Advanced Coatings Based upon Non-Isocyanate
Polyurethanes for Industrial Applications. Surf.
Coat. Int., Part B.

[20] Viada G., Mariotti N., Galliano S., Menozzi A., Barolo C., Bonomo M. (2025). Eco-Friendly and Ready-To-Market Polyurethanes: A Design
of Experiment-Guided Substitution of Toxic Catalyst and Fossil-Based
Isocyanate. ChemSusChem.

[21] Aguiar K. R., Santos V. G., Eberlin M. N., Rischka K., Noeske M., Tremiliosi-Filho G., Rodrigues-Filho U. P. (2014). Efficient
Green Synthesis of Bis­(Cyclic
Carbonate)­Poly­(Dimethylsiloxane) Derivative Using CO 2 Addition: A
Novel Precursor for Synthesis of Urethanes. RSC Adv..

[22] Günther F., Batista Simões M., Imasato H., Pereira
Rodrigues-Filho U. (2019). Experimental and Theoretical Assessment of the Aminolysis
of Cyclo Carbonate to Form Polyhydroxyurethanes. Mater. Today Commun..

[23] Cornille A., Auvergne R., Figovsky O., Boutevin B., Caillol S. (2017). A Perspective
Approach to Sustainable Routes for Non-Isocyanate Polyurethanes. Eur. Polym. J..

[24] Gomez-Lopez A., Grignard B., Calvo I., Detrembleur C., Sardon H. (2020). Monocomponent Non-Isocyanate Polyurethane
Adhesives
Based on a Sol–Gel Process. ACS Appl.
Polym. Mater..

[25] Günther F., Lima E. F. S., Rossi de Aguiar K. M.
F., Bearzi J. R., Simões M. B., Schneider R., Bini R. A., Ribeiro S. J. L., Man M. W. C., Rischka K., Aguiar F. H. B., Pereira R., Mainardi M. D. C. A. J., Rocha M. C., Malavazi I., Passeti T. A., Santos M. L., Imasato H., Rodrigues-Filho U. P. (2020). PDMS-Urethanesil
Hybrid Multifunctional Materials: Combining CO2 Use and Sol–Gel
Processing. J. Sol-Gel Sci. Technol..

[26] Eling, B. ; Friederichs, W. Polyurethanes Polyols, Isocyanates, Rigid Foams, Flexible Foams, Elastomers; De Gruyter, 2025.

[27] Türel T., Cristadoro A. M., Linnenbrink M., Tomović Ž. (2025). Harnessing
Imine Chemistry for the Debonding-on-Demand of Polyurethane Adhesives. ACS Appl. Mater. Interfaces.

[28] Türel T., Schara P., Cristadoro A. M., Linnenbrink M., Tomović Ž. (2025). Acetal-Functionalized Polyurethane
Adhesives: A Path to Debonding-on-Demand. Eur.
Polym. J..

[29] Schara P., Türel T., Pantazidis C., Cristadoro A. M., Sijbesma R. P., Tomović Ž. (2025). Recyclable
Hydrophobic Polyurethanes
and Debondable Coatings Utilizing Apolar Acetal Polyols. ACS Appl. Polym. Mater..

[30] Ates M., Karadag S., Eker A. A., Eker B. (2022). Polyurethane Foam Materials
and Their Industrial Applications. Polym. Int..

[31] Grignard B., Gennen S., Jérôme C., Kleij A. W., Detrembleur C. (2019). Advances in the Use of CO 2 as a
Renewable Feedstock
for the Synthesis of Polymers. Chem. Soc. Rev..

[32] Li, H. ; Zhao, F. ; Cheng, H. CO2-Sourced Polymers: Synthesis, Property, Application Advances in CO2 Utilization. Green Chemistry and Sustainable Technology Zhang, G. ; Bogaerts, A. ; Ye, J. ; Liu, C.-j. Eds. Springer: 2024, 181–207. 10.1007/978-981-99-8822-8_9

[33] Centeno-Pedrazo A., Perez-Arce J., Freixa Z., Ortiz P., Garcia-Suarez E. J. (2023). Catalytic
Systems for the Effective Fixation of CO 2 into Epoxidized Vegetable
Oils and Derivates to Obtain Biobased Cyclic Carbonates as Precursors
for Greener Polymers. Ind. Eng. Chem. Res..

[34] D’Alessandro D. M., Smit B., Long J. R. (2010). Carbon Dioxide Capture: Prospects
for New Materials. Angew. Chem., Int. Ed..

[35] Darensbourg D. J., Holtcamp M. W. (1996). Catalysts for the Reactions of Epoxides and Carbon
Dioxide. Coord. Chem. Rev..

[36] Park, S. E. ; Chang, J. S. ; Lee, K. W. Carbon Dioxide Utilization for Global Sustainability: Studies in Surface Science and Catalysis Proceedings of the 7th International Conference on Carbon Dioxide Utilization, Seoul, Korea, October 12-16, 2003 Elsevier Science 2004 153 606

[37] Malik M., Kaur R. (2018). Synthesis of NIPU by the Carbonation of Canola Oil Using Highly Efficient
5,10,15-Tris­(Pentafluorophenyl)­Corrolato-Manganese­(III) Complex as
Novel Catalyst. Polym. Adv. Technol..

[38] Morales-González M., Valero M. F., Díaz L. E. (2024). Physicochemical and Mechanical Properties
of Non-Isocyanate Polyhydroxyurethanes (NIPHUs) from Epoxidized Soybean
Oil: Candidates for Wound Dressing Applications. Polymers.

[39] Sawpan M. A. (2018). Polyurethanes
from Vegetable Oils and Applications: A Review. J. Polym. Res..

[40] Cai X., Zheng J. L., Wärnå J., Salmi T., Taouk B., Leveneur S. (2017). Influence of Gas-Liquid Mass Transfer on Kinetic Modeling:
Carbonation of Epoxidized Vegetable Oils. Chem.
Eng. J..

[41] Büttner H., Grimmer C., Steinbauer J., Werner T. (2016). Iron-Based Binary Catalytic
System for the Valorization of CO 2 into Biobased Cyclic Carbonates. ACS Sustainable Chem. Eng..

[42] Mazo P. C., Rios L. A. (2012). Improved Synthesis
of Carbonated Vegetable Oils Using
Microwaves. Chem. Eng. J..

[43] Zheng J.-L., Tolvanen P., Taouk B., Eränen K., Leveneur S., Salmi T. (2018). Synthesis of Carbonated
Vegetable
Oils: Investigation of Microwave Effect in a Pressurized Continuous-Flow
Recycle Batch Reactor. Chem. Eng. Res. Des..

[44] Gholami H., Yeganeh H. (2021). Soybean Oil-Derived
Non-Isocyanate Polyurethanes Containing
Azetidinium Groups as Antibacterial Wound Dressing Membranes. Eur. Polym. J..

[45] Boga K., Dhore N. R., Palanisamy A., Patti A. F., Warner J. C., Simon G. P., Saito K. (2023). Bio-Based Light-Healing Isocyanate-Free
Polyurethanes Derived from Carbonated Soybean Oil and Coumarin. Green Chem. Lett. Rev..

[46] Yang X., Wang S., Liu X., Huang Z., Huang X., Xu X., Liu H., Wang D., Shang S. (2021). Preparation of Non-Isocyanate
Polyurethanes from Epoxy Soybean Oil: Dual Dynamic Networks to Realize
Self-Healing and Reprocessing under Mild Conditions. Green Chem..

[47] Rossi
de Aguiar K. M. F., Ferreira-Neto E. P., Blunk S., Schneider J. F., Picon C. A., Lepienski C. M., Rischka K., Rodrigues-Filho U. P. (2016). Hybrid
Urethanesil Coatings for Inorganic Surfaces Produced by Isocyanate-Free
and Sol–Gel Routes: Synthesis and Characterization. RSC Adv..

[48] Pénard A. L., Gacoin T., Boilot J. P. (2007). Functionalized
Sol-Gel Coatings for
Optical Applications. Acc. Chem. Res..

[49] Maver K., Lavrenčič
Štangar U., Judeinstein P., Zanotti J. M. (2008). Dynamic Studies
of Ormosil Membranes. J. Non-Cryst. Solids.

[50] Poussard L., Mariage J., Grignard B., Detrembleur C., Jérôme C., Calberg C., Heinrichs B., De Winter J., Gerbaux P., Raquez J.-M., Bonnaud L., Dubois P. (2016). Non-Isocyanate Polyurethanes from
Carbonated Soybean
Oil Using Monomeric or Oligomeric Diamines To Achieve Thermosets or
Thermoplastics. Macromolecules.

[51] Yang X., Ren C., Liu X., Sun P., Xu X., Liu H., Shen M., Shang S., Song Z. (2021). Recyclable Non-Isocyanate
Polyurethanes Containing a Dynamic Covalent Network Derived from Epoxy
Soybean Oil and CO 2. Mater. Chem. Front..

[52] Pouladi J., Mirabedini S. M., Eivaz Mohammadloo H., Rad N. G. (2021). Synthesis of Novel
Plant Oil-Based Isocyanate-Free Urethane Coatings and Study of Their
Anti-Corrosion Properties. Eur. Polym. J..

[53] Martin-Larrañaga N., Razquin I., Aranburu N., Sanz O., Gonzalez A., Irusta L. (2026). Improving
the Properties of Carbonated Soybean Oil-Based
Non-Isocyanate Polyhydroxyurethane Networks: Copolymerization versus
Hybridization with Epoxy Resin. Prog. Org. Coat..

[54] Zhang W., Wang T., Zheng Z., Quirino R. L., Xie F., Li Y., Zhang C. (2023). Plant Oil-Based
Non-Isocyanate Waterborne Poly­(Hydroxyl
Urethane)­S. Chem. Eng. J..

[55] Liu X., Yang X., Wang S., Wang S., Wang Z., Liu S., Xu X., Liu H., Song Z. (2021). Fully Bio-Based Polyhydroxyurethanes
with a Dynamic Network from a Terpene Derivative and Cyclic Carbonate
Functional Soybean Oil. ACS Sustainable Chem.
Eng..

[56] Bukowczan A., Łukaszewska I., Pielichowski K. (2024). Thermal Degradation
of Non-Isocyanate
Polyurethanes. J. Therm. Anal. Calorim..

[57] Albu P., Bolcu C., Vlase G., Doca N., Vlase T. (2011). Kinetics of
Degradation under Non-Isothermal Conditions of a Thermooxidative Stabilized
Polyurethane. J. Therm. Anal. Calorim..

[58] Parcheta-Szwindowska P., Rohde K., Datta J. (2022). Bio-Derived Polyurethanes Obtained
by Non-Isocyanate Route Using Polyol-Based Bis­(Cyclic Carbonate)­s—Studies
on Thermal Decomposition Behavior. J. Therm.
Anal. Calorim..

[59] Naik A. D., Fontaine G., Bellayer S., Bourbigot S. (2015). Salen Based
Schiff Bases to Flame Retard Thermoplastic Polyurethane Mimicking
Operational Strategies of Thermosetting Resin. RSC Adv..

[60] ASTM International ASTM D1652–11: standard Test Methods for Epoxy Content of Epoxy Resins; ASTM International: West Conshohocken, PA, 2004.

[61] Sitko R., Zawisza B., Malicka E. (2008). Standardless Energy-Dispersive X-Ray
Fluorescence Analysis Using Primary Radiation Monochromatized with
LiF(200) Crystal. Spectrochim. Acta, Part B.

[62] ASTM D543. ASTM D543–21 Testing Plastic Resistance to Chemical Reagents; ASTM International: West Conshohocken, PA, 1999; p. 8.

[63] Kupriyanova G., Smirnov M., Mershiev I., Maraşlı A., Okay C., Mozzhukhin G., Rameev B. (2024). Comparative Analysis
of Vegetable Oils by 1H NMR in Low and High Magnetic Fields. J. Food Compos. Anal..

[64] Bella G., Rotondo A. (2020). Theoretical Prediction of 13C NMR Spectrum of Mixed
Triglycerides by Mean of GIAO Calculations to Improve Vegetable Oils
Analysis. Chem. Phys. Lipids.

[65] Parada
Hernandez N. L., Bonon A. J., Bahú J. O., Barbosa M. I. R., Wolf Maciel M. R., Filho R. M. (2017). Epoxy Monomers Obtained
from Castor Oil Using a Toxicity-Free Catalytic System. J. Mol. Catal. A: Chem..

[66] Farias M., Martinelli M., Bottega D. P. (2010). Epoxidation of Soybean
Oil Using
a Homogeneous Catalytic System Based on a Molybdenum (VI) Complex. Appl. Catal., A.

[67] Miyake Y., Yokomizo K., Matsuzaki N. (1998). Rapid Determination
of Iodine Value
By1H Nuclear Magnetic Resonance Spectroscopy. JAOCS, J. Am. Oil Chem. Soc..

[68] Helbling P., Hermant F., Petit M., Tassaing T., Vidil T., Cramail H. (2023). Unveiling the Reactivity
of Epoxides in Carbonated
Epoxidized Soybean Oil and Application in the Stepwise Synthesis of
Hybrid Poly­(Hydroxyurethane) Thermosets. Polym.
Chem..

[69] Moser B. R., Cermak S. C., Doll K. M., Kenar J. A., Sharma B. K. (2022). A Review
of Fatty Epoxide Ring Opening Reactions: Chemistry, Recent Advances,
and Applications. J. Am. Oil Chem. Soc..

[70] Setien R.
A., Ghasemi S., Pourhashem G., Webster D. C. (2021). Comparison of Epoxidation
Methods for Biobased Oils: Dioxirane Intermediates Generated from
Oxone versus Peracid Derived from Hydrogen Peroxide. Polym. Int..

[71] Nadim E., Paraskar P., Moradi H., Hesabi M., Qiao Y., Murphy E. J., Major I. (2025). Kinetic and Thermodynamic Analysis
of Hemp Oil Epoxidation with Density Functional Theory Insights into
Unsaturated Fatty Acid Epoxidation and Ring-Opening Reactions. Chem. Eng. J. Adv..

[72] Barbosa N. V., dos Santos L. B., Seguchi L. J. E., Silva B. C., de Carvalho T. L., Neto E. T. W., Gimenes R., Soares M. E., Araujo A. J., Silva F. S., Almeida M. R., da Silva M. R. A. (2022). Rapid assessment
of transformer insulating vegetable oil quality using ftir spectroscopy
and chemometrics. Quim. Nova.

[73] Do
Nascimento Filho W. B., Bezerra da Silva H. E., de Cássia Pompeu
de Sousa R., de Souza O. S. (2019). Infrared Spectroscopy FT-IR and X-Ray
Difratometry Applied in the Follow-Up of the Polymerization Process
of Andiroba Oil. Rev. Virtual Quim..

[74] Barreto J., Luna C., Soares N., Souza M., Barros A., Araújo A., Bezerra E., Araújo E., Wellen R. (2025). Epoxidation of Residual
Soybean Oil and Thermal Characterization
of Residual Epoxidized Soybean Oil Crosslinked with Fumaric Acid. J. Polym. Environ..

[75] Sayuti N. S., Ali R., Anuar S. (2021). Synthesis
and characterization of biobased epoxidized
edible oils. Univ. Malaysia Teren. J. Undergrad.
Res..

[76] Destaso F. C., Libretti C., Le Coz C., Grau E., Cramail H., Meier M. A. R. (2025). Optimized Synthesis
of a High Oleic Sunflower Oil Derived
Polyamine and Its Lignin-Based NIPUs. Green
Chem..

[77] Alekseev V. V., Vladimirov S. V., Maklakov L. I., Furer V. L., Furer A. L. (1978). Vibrational
Spectra of Some Urethanes and the Assignment of Bands Related to Urethane
Group Vibrations. J. Appl. Spectrosc..

[78] Carré C., Ecochard Y., Caillol S., Avérous L. (2019). From the Synthesis
of Biobased Cyclic Carbonate to Polyhydroxyurethanes: A Promising
Route towards Renewable Non-Isocyanate Polyurethanes. ChemSusChem.

[79] Türel T., Eling B., Cristadoro A. M., Mathieu T., Linnenbrink M., Tomović Ž. (2024). Novel
Furfural-Derived Polyaldimines
as Latent Hardeners for Polyurethane Adhesives. ACS Appl. Mater. Interfaces.

[80] Camara F., Benyahya S., Besse V., Boutevin G., Auvergne R., Boutevin B., Caillol S. (2014). Reactivity
of Secondary Amines for
the Synthesis of Non-Isocyanate Polyurethanes. Eur. Polym. J..

[81] Cao B., Tang Y., Zhu C., Zhang Z. (1997). Synthesis and Hydrolysis
of Hybridized Silicon Alkoxide: Si­(OEt)­x­(OBut)­4-x. Part I: Synthesis
and Identification of the Si­(OEt)­x­(OBut)­4-X. J. Sol-Gel Sci. Technol..

[82] Peña-Alonso R., Rubio F., Rubio J., Oteo J. L. (2007). Study of the Hydrolysis
and Condensation of γ-Aminopropyltriethoxysilane by FT-IR Spectroscopy. J. Mater. Sci..

[83] Chang, R. ; Goldsby, K. A. Química 11va; McGraw-Hill: Porto Alegre, 2013.

[84] Menczel, J. D. ; Prime, R. B. Thermal Analysis of Polymers, 1 st ed.; Willey: New Jersey, Canada, 2009.

[85] Lind P., Dalene M., Tinnerberg H., Skarping G. (1997). Biomarkers in Hydrolysed
Urine, Plasma and Erythrocytes Among Workers Exposed to Thermal Degradation
Products From Toluene Diisocyanate Foam. Analyst.

[86] Morinaga S., Mori Y., Tada R., Furusho Y., Kawauchi T., Endo T. (2025). Acid-Promoted Depolymerization
of Poly­(Hydroxyurethane)­s to Five-Membered
Cyclic Carbonates Toward Chemical Recycling. ChemSusChem.

[87] Aguirresarobe R. H., Irusta L., Fernandez-Berridi M.
J. (2012). Application of TGA/FTIR
to the Study of the Thermal Degradation Mechanism of Silanized Poly­(Ether-Urethanes). Polym. Degrad. Stab..

[88] Bakkali-Hassani C., Berne D., Ladmiral V., Caillol S. (2022). Transcarbamoylation
in Polyurethanes: Underestimated Exchange Reactions?. Macromolecules.

