# Correction to Polyhydroxyurethane Networks from Soybean Oil and CO_2_: Thermal Behavior, Hybrid Features, and Structural Insights

**DOI:** 10.1021/acsomega.6c03573

**Published:** 2026-05-18

**Authors:** Pedro H. M. Nicácio, Ana Beatriz S. Barros, Carlos B. B. Luna, Andreas Ries, Hidetake Imasato, Edcleide M. Araújo, Ubirajara P. Rodrigues-Filho, Renate M. R. Wellen

## Correction to Acknowledgments and Funding Information

The authors wish to update the Acknowledgments and Funding sections of the original article to include information that was inadvertently omitted during the production stage.

## Final Statement

The authors apologize for any inconvenience caused and confirm that these corrections do not affect the scientific results, discussion, or conclusions of the original article.

