# Supplementary material for: Polyhydroxyurethane Networks from Soybean Oil and CO2: Thermal Behavior, Hybrid Features, and Structural Insights
Source: ACS Omega. 2026 Mar 19;11(12):18715–30. doi: 10.1021/acsomega.5c09377 (PMC13044604; doi:10.1021/acsomega.5c09377)
Supplement: Supplementary file 1 [file ao5c09377_si_001.pdf]

Polyhydroxyurethane Networks from Soybean Oil and CO_2: Thermal Behaviour, Hybrid Features, and Structural Insights

Pedro H. M. Nicácio[1,2], Ana Beatriz S. Barros[1], Carlos B. B. Luna[1], Andreas Ries[3], Hidetake Imasato[2], Edcleide M. Araújo[1], Ubirajara P. Rodrigues-Filho[2], Renate M. R. Wellen[1,4]

[1] Department of Materials Engineering (DEMat), Federal University of Campina Grande (UFCG), 58429-900 Campina Grande, Paraíba, Brazil

[2] Hybrid Materials Chemistry Group (GQMATHI), São Carlos Institute of Chemistry (IQSC), University of São Paulo (USP), 13563-120 São Carlos, São Paulo, Brazil

[3] Bio and Materials Group, Polytechnic School, National University of Asunción, 111421 San Lorenzo, Paraguay

[4] Department of Materials Engineering (DEMAt), Federal University of Paraíba (UFPB), 58051-900 João Pessoa, Paraíba, Brazil

Correspondence should be addressed to: wellen.renate@gmail.com

Supplementary material

ABSTRACT

In response to increasing global demand for bio-based polymers, this study presents the synthesis and comprehensive thermal characterisation of non-isocyanate polyurethanes (NIPUs) derived from soybean oil, focusing on the identification of volatile compounds released during thermal analysis and proposing a degradation mechanism. The materials were obtained via epoxidation, CO_2 cycloaddition, and aminolysis using different amines—IPDA, TRIS, and APTES—forming poly(hydroxyurethanes) (PHUs) with variable crosslink densities and hybrid features. Structural analyses via ^{1}H NMR and FTIR confirmed successful conversion at each step, with epoxy group titration indicating ≈ 91 % conversion to cyclic carbonate and efficient catalyst removal, as confirmed by XRF (Br <0.3% after purification). Gel content tests revealed that TRIS-based PHUs achieved high crosslinking (up to 91.9%), while IPDA-based systems exhibited lower values (71.8%) due to steric hindrance. The presence of APTES significantly modified network architecture, reducing gel content to 33% in some formulations but introducing inorganic siloxane domains. TGA revealed multistage degradation between 250–475 °C, with TRIS-based networks demonstrating enhanced thermal resistance. Deconvoluted DTG curves and TG-IR data identified primary volatiles including CO_2, amines, ketenes, and silanols, elucidating degradation pathways involving urethane cleavage, retro-aminolysis, and glycerol decomposition. This work advances the understanding of structure–property–degradation relationships in NIPUs and highlights their viability as green engineering alternatives to conventional polyurethanes, aligns with SDGs 9, 12, and 13.

Keywords: Non-isocyanate polyurethanes (NIPUs), Poly(hydroxyurethanes) (PHUs), Thermal degradation, CO_2 cycloaddition, Soybean oil-based polymers, Proposing a degradation mechanism.

Table S1. Assignments of the main signals observed in the ^{1}H NMR spectra (400 MHz, $CDCl_3$, 25 °C) of commercial soybean oil (OS), epoxidized soybean oil (ESO), and soybean oil carbonate (CCOS).

Material	Functional acyl groups of related compounds	Structural fragment	δ (ppm)	Relative area*
SO	**Olefinic hydrogens of unsaturated fatty acids**	-CH=CH-	5.3	9.30[(a)]
	**Methine at C_2 of triacylglycerol glycerol**	-CH-OCO-	5.2	1[(b)]
	**Methylenes at C_1 and C_3 of triacylglycerol glycerol**	-CH_2-OCO-	4.1 – 4.4	4[(c)]
	**Bis-allylic methylenes of linoleic and linolenic fatty acids**	-CH=CH-CH_2-CH=CH-	2.7 – 2.8	3.9
	**Methylenes of unsaturated fatty acids/allylic methylenes of mono- and polyunsaturated fatty acids**	CH_2-CH=CH-	2.0 – 2.3	16.3
	**Acyl methylenes of fatty acids**	-CH_2-CH_2-COO-	1.6	6.3
	**Acyl methylenes of fatty acids**	-$(CH_2)_n$-	1.2	54.5
	**Methyls of linolenic acid**	-CH=CH-CH_2-CH_3	1.0	0.7
	**Methyls of fatty acids, except linolenic acid**	-CH_2-CH_3	0.8	8.9
ESO	**Methine at C_2 of triacylglycerol glycerol**	-CH-OCO-	5.3	1[(a)]
	**Methylenes at C_1 and C_3 of triacylglycerol glycerol**	-CH_2-OCO-	4.1 – 4.4	4[(c)]
	**Hydrogens of epoxy groups**	-CHOCH-	2.8 – 3.2	8.4
	**Methylenes between epoxy groups**	-CHOCH-CH_2-CHOCH-	2.2 – 2.3	6
	**Methylenes of long fatty acid chains**	-CH_2-	1.2 – 1.8	77.1
	**Fatty acid methyls**	-CH_2-CH_3	0.8-0.9	8.59
CCOS	**Methine at C_2 of triacylglycerol glycerol**	-CH-OCO-	5.3	0.8[(a)]
	**Hydrogens of cyclic carbonate groups**	-CH-OCOOC-CH-	4.4 – 4.9	4.4
	**Methylenes at C_1 and C_3 of triacylglycerol glycerol**	-CH_2-OCO-	4.1 – 4.4	4[(c)]
	**Hydrogens of remaining epoxy groups**	-CHOCH-	2.8 – 3.2	0.4
	**Methylenes between cyclic carbonates or remaining epoxy groups**	-CH-OCOOC-CH-CH_2-CH-OCOOC-CH- / -CHOCH-CH_2-CHOCH-	2.2 – 2.3	4.6
	**Methylenes of long fatty acid chains**	-CH_2-	1.1 – 2.2	55.6
	**Fatty acid methyls**	-CH_3	0.8-09	7.2

* Experimental data obtained from the analysis of the ^{1}H NMR spectrum of commercial soybean oil and its derivatives

[(a)] Estimated value, subtracting 1 from the integral, normalization factor (FN) for OS, also associated with the methine hydrogen of glycerol; for ESO and CCOS, the calculated value was inserted in the integral, as there are no more Hs from unsaturated bonds, only from glycerol H.

[(b)] Unit value corresponding to the methine proton of glycerol, used as reference.

[(c)] Reference value assuming the glycerol structure.

Calculations performed using ^{1}H NMR spectra

According to Miyake, Yokomizo e Matsuzaki, 1998*(1)*, Farias, Martinelli e Bottega, 2010*(2)*, Parada Hernandez et al., 2017*(3)* e Boerkamp, V. J. P., et al., 2022*(4)* the molar mass (MM) of triglycerides, such

as soybean oil, can be calculated using proton nuclear magnetic resonance (^1H NMR) spectra and can be determined based on Equation S1.

$$MM=\frac{15{,}034*(I+J)}{3*FN}+\frac{14{,}026*(D+E+F+G+H)}{2*FN}+\frac{26{,}016*(A)}{2*FN}+173{,}1 \quad \text{Equation S1}$$

Where FN is the normalization factor of the relative peak area of a proton, based on the signal associated with the four hydrogens of the glycerol methylene group, such that FN is given by Equation S2.

$$FN=\frac{C}{4} \quad \text{Equation S2}$$

The values of A (δ 5.3 ppm), C (δ 4.1–4.3 ppm), D (δ 2.7–2.8 ppm), E (δ 2.2–2.4 ppm), F (δ 1.9–2.1 ppm), G (δ 1.5–1.7 ppm), H (δ 1.2–1.4 ppm), I (δ 0.9–1.3 ppm), and J (δ 0.83–0.93 ppm) in Equations S1 and S2 correspond to the integral areas obtained from the ^1H NMR spectrum.

The iodine value (IV) of commercial soybean oil can be theoretically determined from the average molecular weight and the absolute number of double bonds based on the ^1H NMR spectrum, as shown in Equation S3:

$$IV=\frac{MM_{I_2}*\left(\frac{A}{2}\right)*100}{MM_{SO}} \quad \text{Equation S3}$$

Where MMI_2 is the molar mass of I_2 (253.8 g/mol), A is the integral area of the hydrogens of the unsaturated bonds obtained from ^1H NMR, and MM_{SO} is the molar mass of soybean oil calculated by ^1H NMR.

Due to the presence of groups that are largely inert to the reagents used in the epoxidation of commercial soybean oil, in this work the MM of the soybean oil was not considered; instead, the number of moles of unsaturated bonds in the soybean oil (n_{SO}) was used.

Knowing that the density of commercial soybean oil (ρ_{SO}) at 25 °C is 891 g/L, and using the MMOS obtained from Equation S1, the iodine value from Equation S3, and by deriving the formula for the iodine value (Equation S4), the initial concentration of unsaturated bonds (C_{SO}) present in commercial soybean oil can be determined.

$$IV=\frac{100*m_{I_2}}{m_{SO}}=\frac{100*n_{I_2}*MM_{I_2}}{m_{SO}}=\frac{100*n_{SO}*MM_{I_2}}{\rho_{SO}*V_{SO}}=\frac{100*C_{SO}*MM_{I_2}}{\rho_{SO}} => C_{SO}=\frac{IV*\rho_{SO}}{100*MM_{I_2}} \quad \text{Equation S4}$$

Where mI_2 is the mass of I_2, nI_2 is the number of moles of I_2, and m_{SO} is the mass of soybean oil.

To quantify the yields of epoxidation and cyclocarbonation by ^1H NMR, the following equations S5, S6 and S7 were used:

$$Epoxidation\ (\%)=\frac{\left(\frac{\left(\frac{C_{ESO}}{Np_{ESO}}\right)}{\left(\frac{B_{ESO}}{Np_{ref}}\right)}\right)}{\left(\frac{\left(\frac{A_{SO}}{Np_{SO}}\right)}{\left(\frac{C_{SO}}{Np_{ref}}\right)}\right)}*100 \quad \text{Equation S5}$$

$$EEW=\frac{\left(\frac{C_{ESO}}{Np_{ESO}}\right)}{\left(\frac{B_{ESO}}{Np_{ref}}\right)} \quad \text{Equation S6}$$

$$Cyclocarbonation\ conversion\ rate\ (\%)=\frac{(B_{CSBO})}{(B_{CSBO}+D_{CSBO})}*100 \quad \text{Equation S7}$$

Where A_{SO} and C_{SO} are the integral areas of the regions δ 5.3–5.4 and 2.7–2.8 ppm in the ^{1}H NMR spectrum of soybean oil, N_{PSO} is the number of protons corresponding to a double bond present in the soybean oil, N_{Pref} is the number of methylene protons present in the glycerol molecule, B_{ESO} and C_{ESO} are the integral areas of the regions δ 4.1–4.4 and 2.8–3.2 ppm in the ^{1}H NMR spectrum of the epoxidized soybean oil, N_{PESO} is the number of protons corresponding to an oxirane ring present in the epoxidized soybean oil, and B_{CSBO} and D_{CSBO} are the integral areas of the regions δ 4.4–4.9 and 2.7–2.8 ppm of the CSBO.

Calculations performed based on titration data

The titration endpoint is indicated by the color change of the indicator, initially purple, then blue, and finally green, allowing the determination of the epoxy content (E), epoxy equivalent weight (EEW), and the indirect cyclocarbonation yield—i.e., estimated based on the quantification of residual epoxide present in the cyclocarbonate—calculated using the equations S8, S9 and S10

$$E=4.3*V*(\frac{N}{W}) \quad \text{Equation S8}$$

$$W_{EEW}=43*(\frac{100}{E}) \quad \text{Equation S9}$$

$$CSBO\ \%=\left(\frac{E_{ESO}\text{-}E_{CSBO}}{E_{ESO}}\right)*100 \quad \text{Equation S10}$$

Where N is the normalization factor of perchloric acid (0.12), W is the mass of the titrated material in grams, V is the volume of perchloric acid required for the titration in milliliters, E is the epoxy weight percentage, W_{EEW} is the epoxy equivalent weight, and CSBO % is the indirect cyclocarbonation yield.

Catalyst extraction by liquid–liquid washing

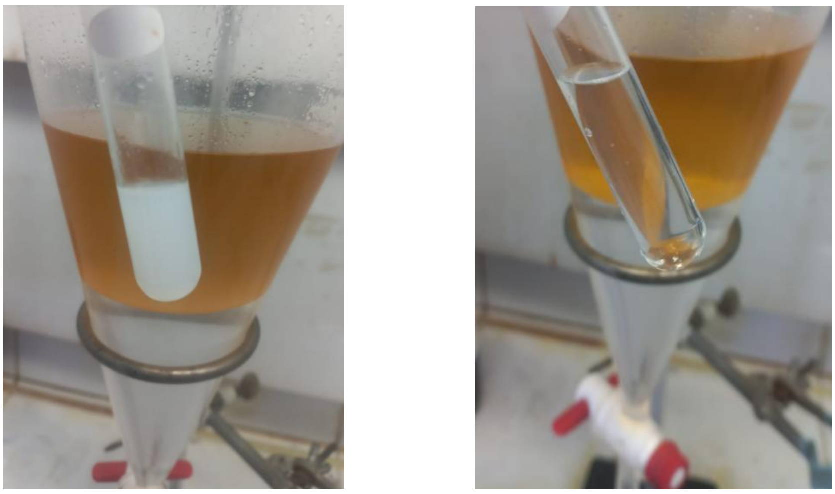

(a) (b)

Figure S1. Qualitative tests performed with $AgNO_3$ during the first (a) and sixth (b) steps of the liquid–liquid extraction.

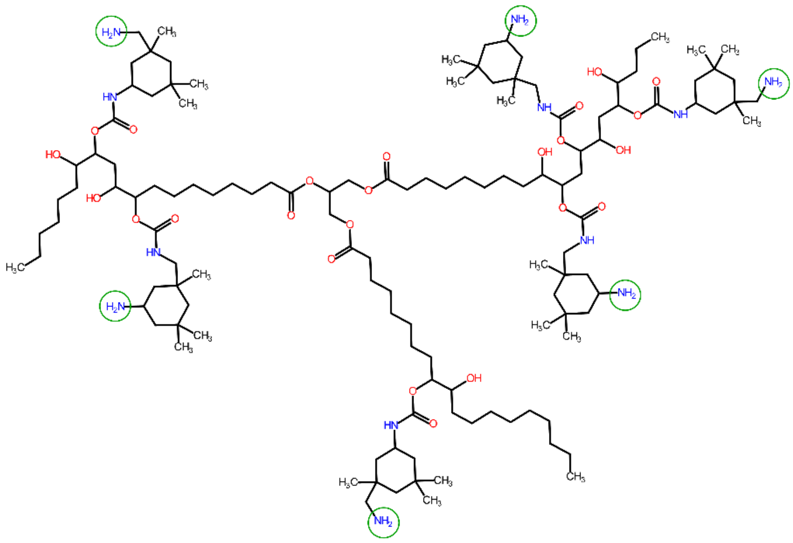

(a)

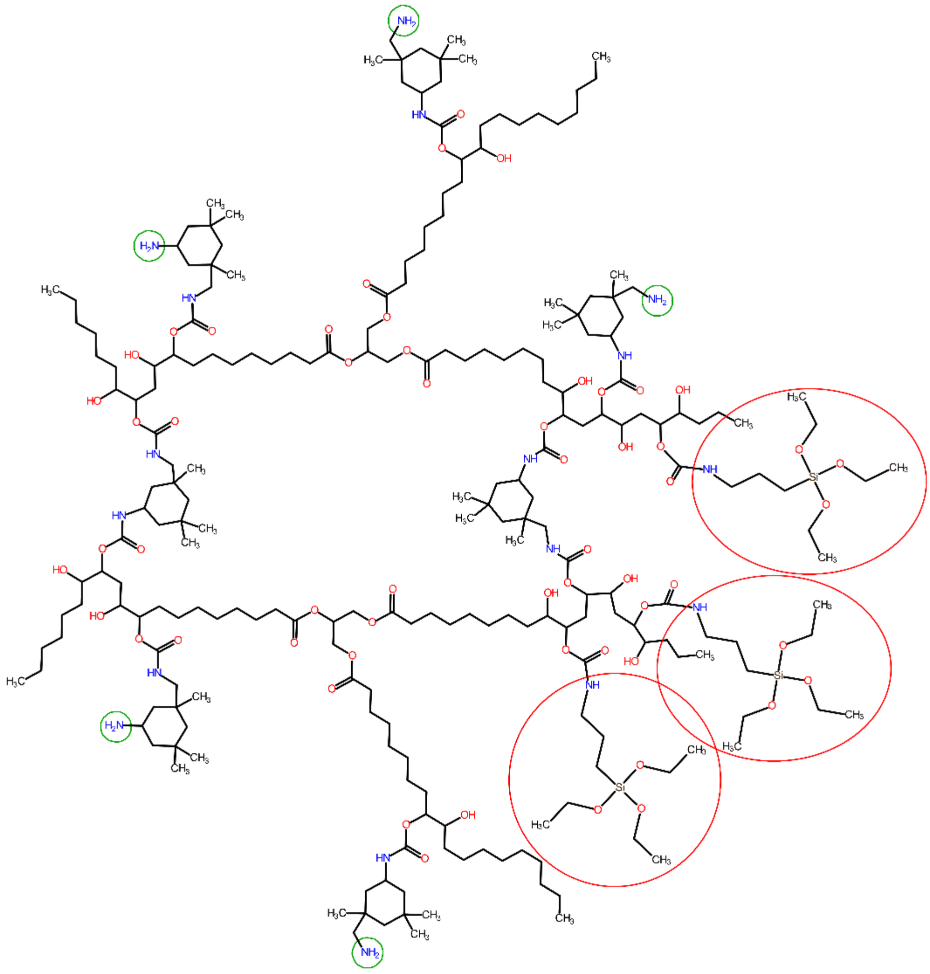

(b)

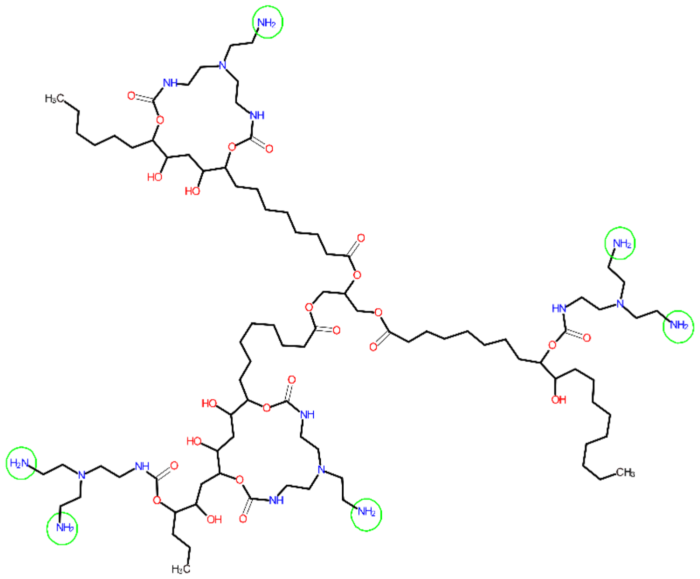

(c)

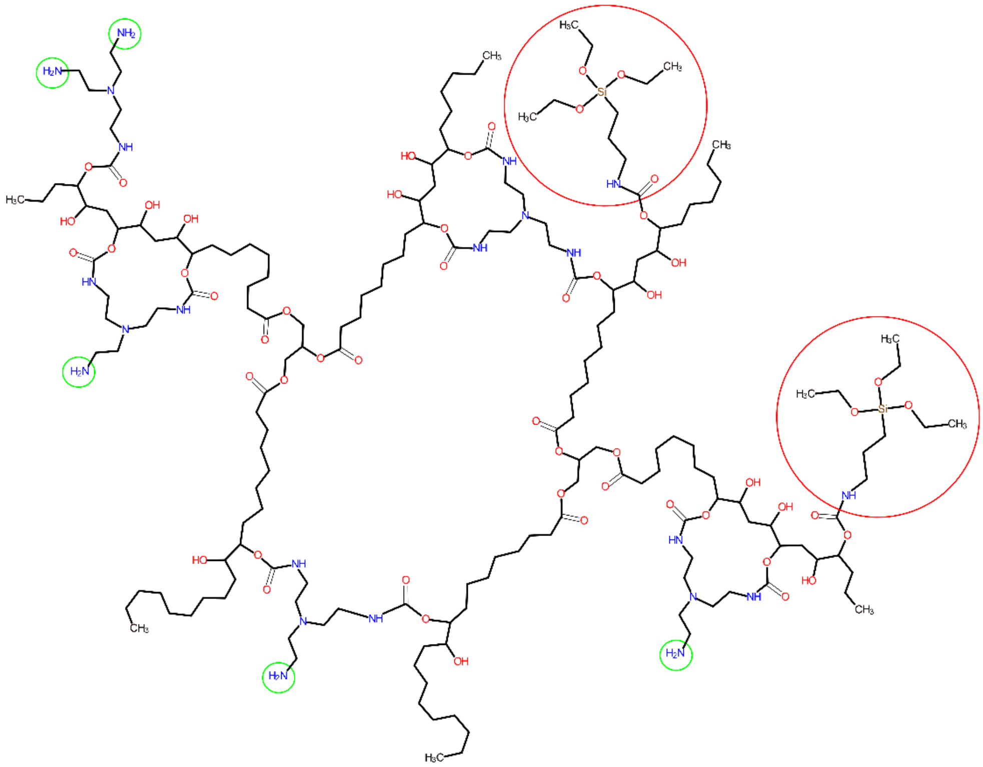

(d)

Figure S2. Proposal for the reticulated structures for CSBO/IPDA (a), CSBO/IPDA/APTES (b), CSBO/TRIS (c) and CSBO/TRIS/APTES (d). The green circles represent primary amine groups capable of reacting with cyclocarbonate moieties from other oil molecules, whereas the red circles indicate the ethoxysilane domains of APTES, which can form silanols through interactions with the hydroxyl groups present in the PHUs.

The acquisition of the DTG deconvolution data was performed using Gaussian integration (Equation S11), as shown in Figure S3.

$$y=y_0+\frac{A*e^{\frac{-4*ln(2)*(x-x_c)^2}{w^2}}}{w\sqrt{\frac{\pi}{4*ln(2)}}} \qquad \text{Equation S11}$$

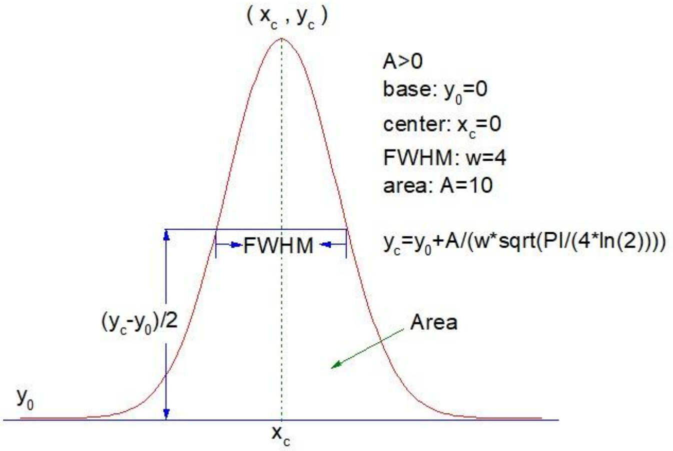

Figure S3. Data acquisition model for a deconvoluted peak.

Where y is the height at each point of the peak, y_0 is zero, A is the peak area, y_c is the maximum peak height, x is the peak temperature at a given point, x_c is the central temperature at which the peak reaches its maximum height, and w is the full width at half maximum (FWHM).

Table S2. Data obtained from the Gaussian deconvolution peaks at a heating rate of 10 °C·min^{-1} for all formulations investigated.

Event	Formulation	Collected Data		
		A	FWHM	T_p (°C)
**Water loss**	**CSBO/IPDA**	9.71	48.01	122.18
	**CSBO/IPDA/APTES**	8.96	46.95	110.51
	**CSBO/TRIS**	11.09	43.81	120.18
	**CSBO/TRIS/APTES**	8.19	41.44	113.79
**Free primary amine degradation**	**CSBO/IPDA**	45.81	63.01	242.20
	**CSBO/IPDA/APTES**	46.86	46.95	243.29
	**CSBO/TRIS**	46.71	58.34	250.77
	**CSBO/TRIS/APTES**	47.35	54.44	257.16
**First stage in the degradation with low degree of cross-linked of PHUs**	**CSBO/IPDA**	352.04	79.93	325.03
	**CSBO/IPDA/APTES**	280.79	72.07	318.19
	**CSBO/TRIS**	142.35	52.22	300.03
	**CSBO/TRIS/APTES**	169.91	55.24	314.98
**Second stage in the degradation with low degree of cross-linked of PHUs**	**CSBO/IPDA**	281.65	36.50	371.31
	**CSBO/IPDA/APTES**	308.82	43.32	371.82
	**CSBO/TRIS**	153.24	38.33	339.97
	**CSBO/TRIS/APTES**	144.33	39.79	347.18
**First stage in the degradation with high degree of cross-linked of PHUs**	**CSBO/IPDA**	143.74	28.88	417.36
	**CSBO/IPDA/APTES**	195.78	46.95	418.58
	**CSBO/TRIS**	252.36	36.68	384.98
	**CSBO/TRIS/APTES**	330.49	46.75	403.38
**Second stage in the degradation with high degree of cross-linked of PHUs**	**CSBO/IPDA**	85.64	28.29	445.97
	**CSBO/IPDA/APTES**	17.28	17.13	450.01
	**CSBO/TRIS**	173.32	39.67	435.01
	**CSBO/TRIS/APTES**	124.65	27.90	448.06
**Carbonization of degradation produts**	**CSBO/IPDA**	112.27	88.90	538.76
	**CSBO/IPDA/APTES**	148.50	89.81	532.73
	**CSBO/TRIS**	191.89	101.99	543.65
	**CSBO/TRIS/APTES**	141.60	97.45	544.99
**FIT of the Plot**	**Formulation**	**R^2**		

**FIT**	**CSBO/IPDA**	0.99579
	**CSBO/IPDA/APTES**	0.99044
	**CSBO/TRIS**	0.99795
	**CSBO/TRIS/APTES**	0.99595

A = area; FWHM = Width at half height of the peak; T_p = peak temperature; R^2 = Correlation coefficient

Referencias

1. Miyake, Y.; Yokomizo, K.; Matsuzaki, N. Rapid determination of iodine value by1H nuclear magnetic resonance spectroscopy. *JAOCS, Journal of the American Oil Chemists' Society* **1998**, *75*(1), 15–19. doi:10.1007/s11746-998-0003-1.

2. Farias, M.; Martinelli, M.; Bottega, D. P. Epoxidation of soybean oil using a homogeneous catalytic system based on a molybdenum (VI) complex. *Applied Catalysis A: General* **2010**, *384*(1–2), 213–219. doi:10.1016/j.apcata.2010.06.038.

3. Parada Hernandez, N. L.; Bonon, A. J.; Bahú, J. O.; Barbosa, M. I. R.; Wolf Maciel, M. R.; Filho, R. M. Epoxy monomers obtained from castor oil using a toxicity-free catalytic system. *Journal of Molecular Catalysis A: Chemical* **2017**, *426*, 550–556. doi:10.1016/j.molcata.2016.08.005.

4. Boerkamp, V. J. P.; Merkx, D. W. H.; Wang, J.; Vincken, J.-P.; Hennebelle, M.; van Duynhoven, J. P. M. Quantitative assessment of epoxide formation in oil and mayonnaise by 1H-13C HSQC NMR spectroscopy. *Food Chemistry* **2022**, *390*, 133145. doi:10.1016/j.foodchem.2022.133145.
